# Self-assembled amphiphilic fluorescent probe: detecting pH-fluctuations within cancer cells and tumour tissues†

**DOI:** 10.1039/d0sc03795h

**Published:** 2020-08-28

**Authors:** Soo Yeon Kim, Arup Podder, Hyunseung Lee, Youn-Joo Cho, Eun Hee Han, Sabina Khatun, Jonathan L. Sessler, Kwan Soo Hong, Sankarprasad Bhuniya

**Affiliations:** Research Center for Bioconvergence Analysis, Korea Basic Science Institute Cheongju 28119 Korea kshong@kbsi.re.kr; Amrita Centre for Industrial Research & Innovation, Amrita Vishwa Vidyapeetham Ettimadai Coimbatore 641-112 India b_sankarprasad@cb.amrita.edu; Graduate School of Analytical Science and Technology, Chungnam National University Daejeon 34134 Korea; Department of Chemistry, The University of Texas at Austin Austin Texas 78712-1224 USA sessler@cm.utexas.edu; Centre for Interdisciplinary Science, JIS Institute of Advanced Studies and Research, JIS University Kolkata 700-091 India

## Abstract

Abnormal anaerobic metabolism leads to a lowering of the pH of many tumours, both within specific intracellular organelles and in the surrounding extracellular regions. Information relating to pH-fluctuations in cells and tissues could aid in the identification of neoplastic lesions and in understanding the determinants of carcinogenesis. Here we report an amphiphilic fluorescent pH probe (**CS-1**) that, as a result of its temporal motion, provides pH-related information in cancer cell membranes and selected intracellular organelles without the need for specific tumour targeting. Time-dependent cell imaging studies reveal that **CS-1** localizes within the cancer cell-membrane about 20 min post-incubation. This is followed by migration to the lysosomes at 30 min before being taken up in the mitochondria after about 60 min. Probe **CS-1** can selectively label cancer cells and 3D cancer spheroids and be readily observed using the green fluorescence channel (*λ*_em_ = 532 nm). In contrast, **CS-1** only labels normal cells marginally, with relatively low Pearson's correlation coefficients being found when co-incubated with standard intracellular organelle probes. Both *in vivo* and *ex vivo* experiments provide support for the suggestion that **CS-1** acts as a fluorescent label for the periphery of tumours, an effect ascribed to proton-induced aggregation. A much lower response is seen for muscle and liver. Based on the present results, we propose that sensors such as **CS-1** may have a role to play in the clinical and pathological detection of tumour tissues or serve as guiding aids for surgery.

## Abstract 2

A self-assembled amphiphilic fluorescent probe allows pH-fluctuations within cancer cells and tumour tissues to be readily detected.

## Graphical abstract

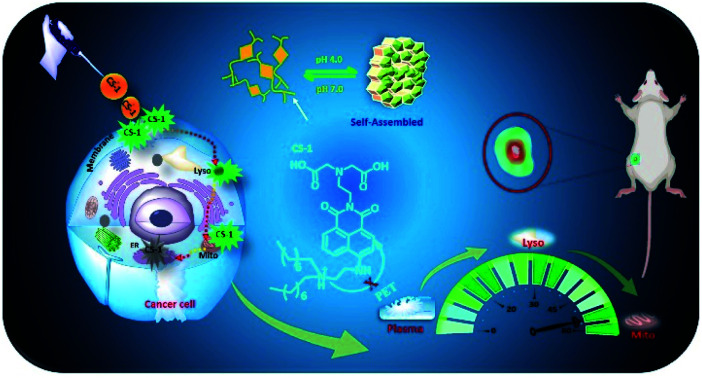

## Introduction

In general, tumorigenic cells are not self-sufficient. Cancer cells take support from healthy cells, the extracellular matrix, and various cell-communication entities so as to be able to grow even under anaerobic conditions.^1^ As a consequence, the tumour microenvironment becomes heterogeneous, which creates a barrier against treatment and diagnosis.^1^ Anaerobic glycolysis in cancer cells metabolizes glucose to lactic acid instead of pyruvate as usually occurs in healthy cells.^2^ This results in cancerous cells becoming somewhat acidic, a change that translates to various intracellular organelles and the extracellular membrane. In addition to the reduction in pH arising from the build-up of lactic acid, the excess CO_2_ produced as the result of increased metabolic activity is converted to carbonic acid *via* carbonic anhydrase, thus providing a source of H^+^.^3^ Along with increased anaerobic glycolysis in mitochondria, rapid oxidative phosphorylation in mitochondria is associated with acidosis in tumour cells.^4,5^ The excess protons (H^+^) are then pumped to the region of the outer membrane so as to maintain the inner mitochondrial membrane potential (*Ψ*, MIM) (−145 mV) at a level commensurate with ATP synthesis. As a consequence, the outer membrane of mitochondria in tumour cells is typically slightly acidic (pH ≤ 6.8).^6^

The acidosis characteristic of tumours, generally referred to as the Warburg effect, is something that can cause the immune system to mis-identify cancerous tissues and cells.^7^ Moreover, acidosis provides an environment conducive to cancer-abetting proteases and tumour angiogenesis.^8^ The precise mapping of pH fluctuations and organelle-level pH differences might allow for the identification of cancers. To date, non-invasive magnetic resonance imaging has been used to map pH levels in tumours *in vivo*.^9^ However, pH-dependent MR imaging is generally unable to provide spatiotemporal images with super-high resolution. Fluorescence imaging represents an attractive alternative since it can provide, at least in principle, high resolution spatiotemporal images while perturbing only minimally the system as a whole.

Recently, a large number of small molecular fluorescent probes have been developed for studying intracellular pH levels in live cells.^10–14^ However, to our knowledge and without exception, these systems have required conjugation to specific targeting entities. For instance, Kim *et al.* developed a mitochondria targeted fluorescent probe that can monitor pH fluctuations under conditions of artificial mitophagy and nutrient starvation.^15,16^ Separately, nanomaterials^16^ and quantum dots,^17^ as well as polymer-, peptide- and DNA-based materials,^18–21^ have been used to estimate the pH of living cells. Cell surface pH mapping^22^ has also been achieved using DNA,^23^ peptide,^24^ and phospholipid^25^ conjugated fluorescent probes. A monoclonal antibody trastuzumab-conjugated, bodipy-based pH probe that localizes within the lysosomes of cancer cells and allows discrimination between cancerous and non-cancerous cells was reported by Urano *et al.*^26^ A Cy7-dye conjugated integrin receptor (ABIR)^27^ has likewise been used for mapping the pH of lysosomes in cancer cells. Han *et al.* reported an aza-bodipy dye for the labelling of tumours that is internalized through cell surface binding to a tumour membrane-targeting ligand peptide (pHLIP).^28^ Recently, Urano *et al.* developed a Si-rhodamine fluorescence probe for pH mapping in live cells.^29^ These researchers estimated apparent pH fluctuations in cellular organelle through time-lapse imaging using transferrin tagged Si-rhodamine. Later, this same group reported a hydroxyl methyl germanium-rhodamine (HMGeR) derivative that acts as a NIR-fluorescent probe and which could be tagged with avidin for pH mapping of vesicles during endocytosis, as well as tumour tissues.^30^

As a general rule, the retention times of known small molecule fluorescent probes inside cells is relatively low.^31^ However, several pH probes have been internalized into cancer cells as the result of a tumour-targeting entity and used for the selective labelling of cancer cells or activate chemotherapeutics agents in cancer cells, such as HIα1,^32,33^ NQO1,^34,35^ H_2_S,^36,37^ and others.^38,39^ However, these probes are specific to special of cancer cell phenotypes. More broadly, an ability to map the pH of cancer cells and organelles remains a challenge. This difficulty is compounded by the fact that the effective local proton concentrations can change quickly. Moreover, the broad diversity of cancer as a disease, renders challenging, and perhaps impractical, current strategies involving creating individual biomarker-conjugated probes. What is needed is a fluorescent pH sensing probe with broad applicability.

We hypothesized that proton (H^+^) driven self-assembly of an otherwise non-fluorescent probe may allow insights into pH fluctuations in cancer cells without the need for conjugation to a targeting entity. In this study, we present a long-chain amphiphilic pH probe (**CS-1**) that acts as a fluorescent pH sensor; it may be used to discriminate normal cells and tissues from cancerous ones while allowing for the qualitative monitoring of pH fluctuations at the membrane surface and within various organelles of both cancerous and non-cancerous cells. Ultimately, such probes could have a role to play in the diagnosis and studying of cancerous diseases while providing potentially new and useful research tools.

## Results and discussion

### Synthesis and photophysical properties of probe **CS-1**

**Scheme 1:**
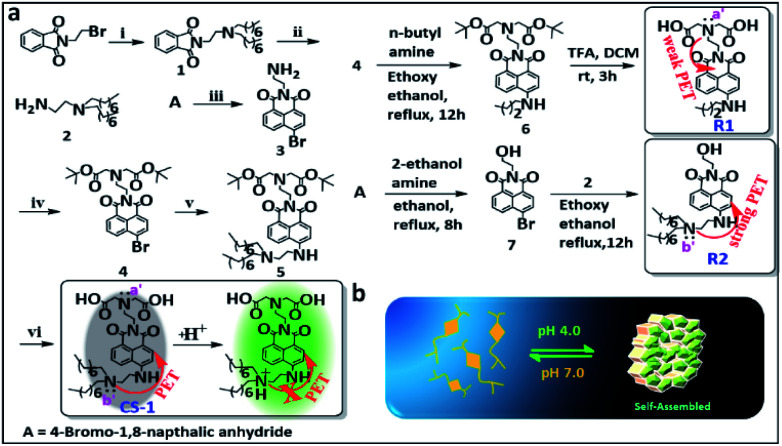
(a) Synthesis of probe **CS-1**. Reagents: (i) *n*-dioctylamine, K_2_CO_3_, acetonitrile (ACN); at 70 °C for 12 h. (ii) NH_2_NH_2_·H_2_O, ACN; at RT for 12 h. (iii) ethylenediamine, EtOH; at RT for 2 h. (iv) *tert*-butyl bromoacetate, K_2_CO_3_, ACN; at 70 °C for 12 h. (v) Compound **2**, ethoxy ethanol for 16 h; reflux at 140 °C. (vi) Trifluoroacetic acid (TFA), CH_2_Cl_2_, EtOH; at RT for 3 h. (b) Schematic view of the proposed acid-promoted self-assembly of probe **CS-1**.

The amphiphilic long-tail pH probe **CS-1** was designed to allow the mapping of pH levels in different organelles in cancer cells. Its synthesis is summarized in Scheme 1. Briefly, **CS-1** was synthesized in six successive steps. First, compound **2** was obtained from **A** in two steps in good yield. Separately, 8-bromonaphthalic anhydride was reacted with ethylene diamine to give compound **3**.^40^ Compound **3** was subject to N-alkylation with *tert*-butyl bromoacetate to provide intermediate **4**. Reaction of **4** with **2** yielded compound **5**. Finally, the *tert*-butyl group present in **5** was deprotected by treatment with TFA giving the desired long-tail amphiphilic pH probe, **CS-1**. Two reference compounds, **R1** and **R2**, were prepared so as to explore the role of the amino centres labelled as N (a′/b′) in controlling the putative pH-dependent fluorescence response. The synthesis of compounds **1–7**, **CS-1**, **R1**, and **R2** is described in the Materials and Methods section. ^1^H NMR and ^13^C NMR spectra and HR-MS data for each new compound are provided in the ESI (Fig. S1–S27†). We chose naphthalimide as the core fluorophore in the present study because it is relatively photostable and rather chemically inert.^15,35,41^

With probe **CS-1** in hand, we studied its ability to detect pH fluctuations in aqueous solution. This was done by recording its UV-Vis spectrum as a function of pH. It was found that in aqueous media the UV-absorption intensity of **CS-1** at 460 nm gradually increases as the pH is lowered from 8.5 to 4.0 (Fig. S28†). The fluorescence intensity of **CS-1** at 531 nm was also found to increase as the pH of the buffer solution was lowered (Fig. S29†). More specifically, the fluorescent spectral intensity of **CS-1** was found to be 8-fold greater at pH 4.0 than at pH 8.5. The quantum yield under identical conditions increased from 0.243 (pH 8.5) to 0.901 (pH 4.0) (Fig. S30†). Little interference in the response was seen when probe **CS-1** was subject to changes in pH in the presence of other biological analytes (Fig. S31a†).

This lack of interference coupled with the pH-dependent reversibility of the fluorescence signal of **CS-1** (Fig. S31b†) led us to consider that **CS-1** might prove suitable for mapping pH fluctuations *in vitro*. The calculated pKa value for **CS-1** (5.99 ± 0.09) provided a further incentive to test whether it could be used a pH probe that would provide meaningful information regarding the acidic environment characteristic of cancer cells (Fig. S32†). Probe **CS-1** has two tertiary-nitrogen atoms (labelled as N a′,b′ in Scheme 1), at least one of which could operate as a PET-donor to suppress the fluorescence intensity **CS-1** in alkaline pH. In other words, in acidic medium, protonation of the nitrogen donors was expected to nullify the PET effect,^16,42–44^ leading to a restoration of the fluorescence. Support for this proposition comes from studies of the reference compound **R1** and **R2** as a function of pH (range: pH 8.5 → 4.0). As can be seen from an inspection of Fig. S33a,† the fluorescence intensity of **R2** increases as the pH is lowered; in contrast, the fluorescence intensity of **R1** changes only slightly over the 8.5 → 4.0 pH range (Fig. S33b†). From these experimental observations, we conclude that the tertiary amine bearing a long-chain (b′) plays a significant role in regulating the fluorescence response of **CS-1** to variations in pH.

### Cell labelling and intracellular localization studies

**Fig. 1:**
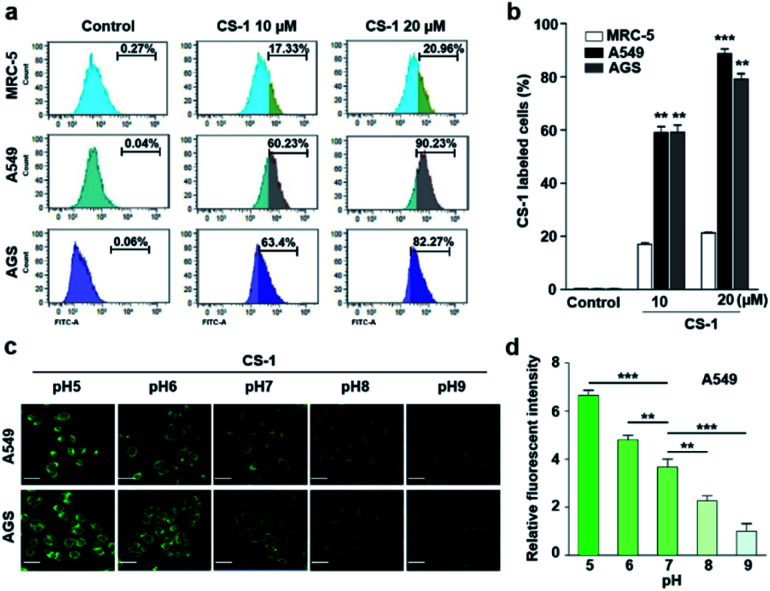
**CS-1** labelling study in cancer (A549, AGS) and normal (MRC-5) cells. (a and b) Quantitative cell analyses of **CS-1**-labeled cells by flow cytometry. Cells, incubated at ∼pH 7.4 in phosphate-buffered saline, were treated with **CS-1** (10 and 20 μM) for 30 min prior to carrying out confocal laser scanning microscopic (CLSM) imaging to determine the intracellular distribution of **CS-1**. (c) Representative CLSM images of cancer (A549 and AGS) cells incubated with **CS-1** (20 μM) for 30 min in cell culture media of differing pH (*i.e.*, 5, 6, 7, 8, and 9), and (d) a quantitative comparison of the fluorescence intensity *vs.* pH. Experiments were repeated at least 3 times. Graphs show mean ± SD. *P < 0.05, **P < 0.01, and ***P < 0.001 (two-tailed unpaired Mann–Whitney).

In an effort to test whether probe **CS-1** could be used to discriminate cancer cells from normal cells by mapping pH fluctuations, fluorescence imaging studies were carried out using A549 (human lung adenocarcinoma), AGS (human gastric cancer), and MRC-5 human normal lung fibroblastic cells. Following confirmation that the cytotoxicity of **CS-1** was not appreciable up to the 100 μM level (Fig. S34†), both sets of cells were treated with **CS-1** in a dose-dependent manner. The acquired fluorescence images revealed that the fluorescence intensity in the A549 cells was greater than in the MRC-5 cells by a factor of approximately 3 (*cf.* Fig. S35†). Based on flow cytometric analyses (Fig. 1a, b), it was concluded that A549 and AGS cancer cells were labelled at the 90% and 82% level, respectively, when treated with **CS-1** (20 μM). In contrast, MCR-5 normal cells were only labelled at the 20% level under similar conditions. These results are consistent with the design expectation that probe **CS-1** would be able to discriminate cancer cells from normal cells based on extracellular/intracellular pH variations and do so without the need for a specific tumour-targeting ligand.

In a time-dependent study, we noticed that the extent of cancer cell labelling increased with time after treating with **CS-1** (Fig. S35c and d†). This finding was ascribed to acidosis. To provide support for this postulate, A549 cells were treated with **CS-1** while being maintained at different pH (5.0 → 9.0). The fluorescence intensity of the cells treated with **CS-1** was found to decrease as the pH of the cell medium increased (Fig. 1c and d). This was taken as evidence that probe **CS-1** might serve as a marker for acidosis.

The intracellular localization of probe **CS-1** in A549 cancer and MRC-5 normal cells was evaluated through co-staining with various organelle-specific trackers for lysosome, mitochondria, plasma membrane, and endoplasmic reticulum, respectively. The extent of fluorescence overlap for **CS-1** with various organelles was codified in the form of the Pearson's overlap coefficient (PC) averaged over at least 3 independent experiments. The overlap was found to be higher in the acidic compartments of A549 cancer cells, such as lysosomes (PC = 0.70 ± 0.05), mitochondria (PC = 0.73 ± 0.02), and plasma membrane (PC = 0.44 ± 0.05), than in MRC-5 normal cells (PC = 0.18 ± 0.03, 0.10 ± 0.04, and 0.05 ± 0.04, respectively) (*cf.* Fig. S36†). In contrast, the PC values for **CS-1** in the endoplasmic reticulum in both the cancer and normal cells proved to be essentially the same, and very low compared with other organelles (*i.e.*, PC = 0.03 ± 0.02 and 0.07 ± 0.02 in cancer and normal cells, respectively). This latter result is ascribed to the relatively high pH (≥7.3) of the endoplasmic reticulum in both cancer and normal cells.^45^ Taken together, these results lead us to suggest that by monitoring appropriate organelles, probe **CS-1** could be used to distinguish between normal and cancerous cell lines.

### Dynamic changes in the intra-cellular localization of **CS-1** and relative pH mapping in live cells

**Fig. 2:**
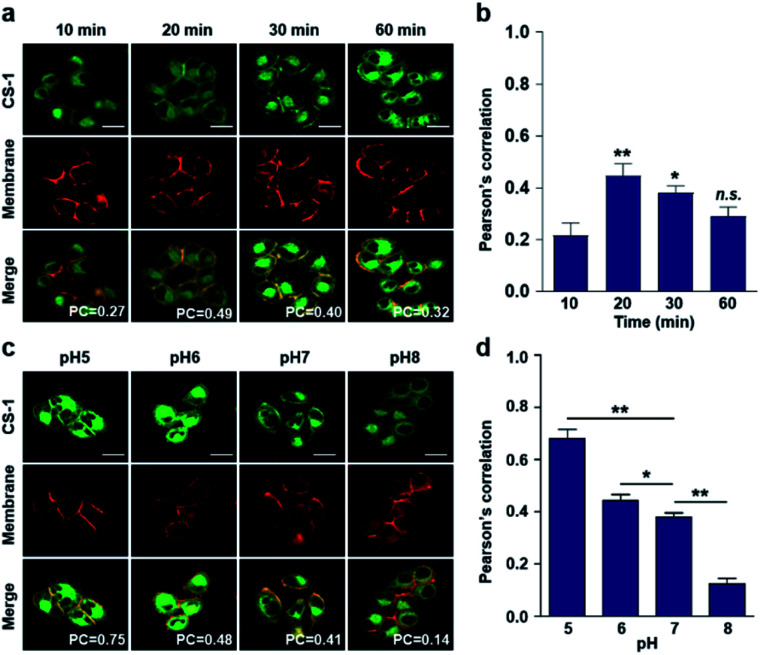
Time- and pH-dependent colocalization of **CS-1** and plasma membrane in A549 cancer cells. (a and b) A549 cells, incubated at ∼pH 7.4, were treated with **CS-1** (10 μM) for 10, 20, 30 min and 1 h, together with a plasma membrane stain (CellMask™, 250 nM) for 30 min. (a) Representative CLSM images for **CS-1** (green) and the membrane stain (red); study replicated in 3 independent experiments. (b) Corresponding Pearson's correlation coefficients (PC) between **CS-1** and the membrane tracker. (c and d) A549 cells were treated with **CS-1** (10 μM) and the membrane stain and incubating for 30 min in cell medium of pH of 5, 6, 7, and 8. (c) Representative CLSM images for **CS-1** and the membrane stain; study replicated in 3 independent experiments. (d) Corresponding PC values plotted as a function of pH. Scale bar: 20 μm. Graphs show mean ± SD. *P < 0.05 and **P < 0.01 (two-tailed unpaired Mann–Whitney).

Typically, exogenous organic molecules enter cells *via* endocytosis; subsequently, they pass through the cell membrane to lysosomes before further localizing to other organelles.^46^ We were thus interested in determining whether such expected redistributions would allow **CS-1** to be used as a temporal, organelle-specific pH probe within live cells. Toward that end, time-dependent fluorescence images were acquired in conjunction with various organelle-specific trackers. Even at its highest, the overlap coefficient with the plasma membrane tracker proved to be moderate (maximum PC = 0.45 ± 0.05 at 20 min), with a decrease as a function of time being seen after the initial rise (Fig. 2a and b). To establish a potential relationship between the overlap coefficient and the plasma pH, we determined the overlap coefficient of probe **CS-1** with a known plasma membrane marker (CellMask™) as a function of pH. For this, we varied the cellular pH artificially from 5 to 8, and then measured the overlap coefficient. We noticed that the value of the overlap coefficient decreased with increasing plasma pH and in a linear fashion (Fig. 2c and d). This critical observation led us to surmise that the plasma pH could be estimated by determining the overlap coefficient of probe **CS-1** with a standard plasma membrane marker.

In accord with the above design expectation, it was found that the value of the colocalization coefficient in the plasma at 20 min (PC = 0.45 ± 0.05, Fig. 2b) for cells incubated at 7.4 is similar to that seen for the pH 6 control studies (PC = 0.44 ± 0.04) (Fig. 2d). We take this as evidence that when **CS-1** is used in conjunction with a plasma membrane tracker the apparent pH value of the plasma membrane can be readily assessed 20 min post-incubation. Importantly, the inferred pH value accords with the literature, namely that the plasma pH of cancer cells varies between 6.0 and 6.8.^22^

**Fig. 3:**
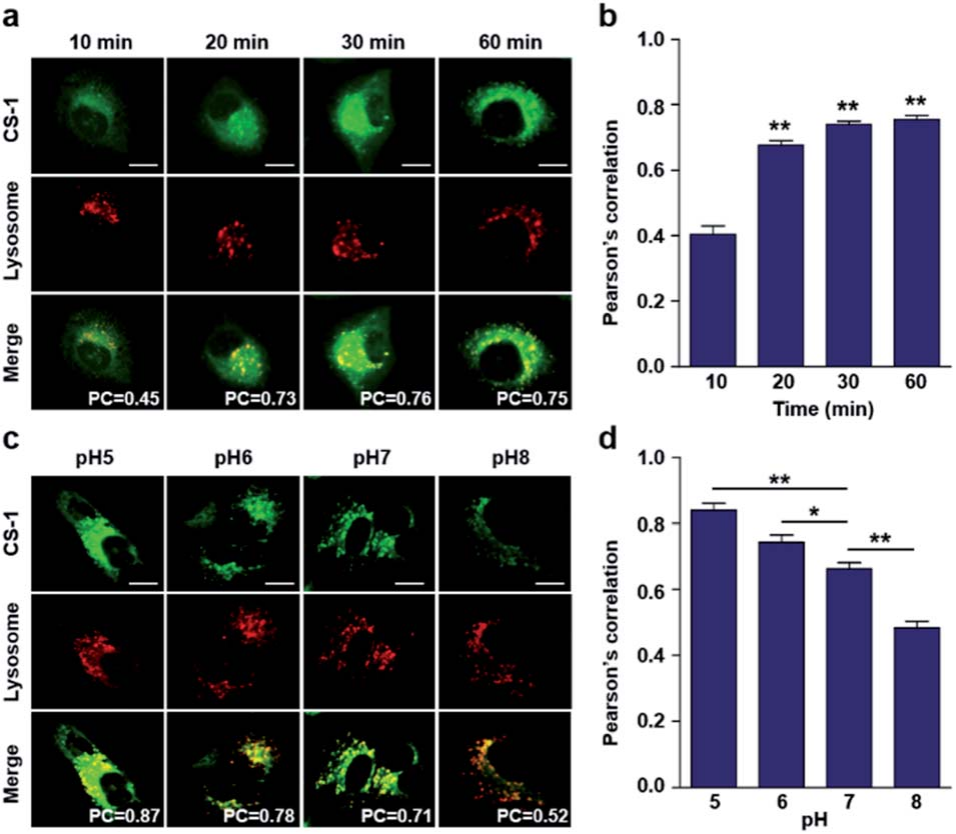
Time- and pH-dependent colocalization of **CS-1** and a lysosomal stain in A549 cancer cells. (a and b) A549 cells, incubated at ∼pH 7.4, were treated with **CS-1** (10 μM) for 10, 20, 30 min and 1 h, followed by staining with LysoTracker® Red DND-99 (100 nM) for 30 min. (a) Representative CLSM images for **CS-1** and LysoTracker®, and (b) corresponding Pearson's correlation coefficients (PC) averaged over 3 independent experiments. (c and d) A549 cells were incubated with **CS-1** (10 μM) and LysoTracker® for 30 min in cell culture medium of pH 5, 6, 7, and 8. (c) Representative CLSM images for **CS-1** and LysoTracker®, and (d) corresponding PC values as a function of pH; study replicated in 3 independent experiments. Scale bar: 20 μm. Graphs show mean ± SD. *P < 0.05 and **P < 0.01 (two-tailed unpaired Mann–Whitney).

**Fig. 4:**
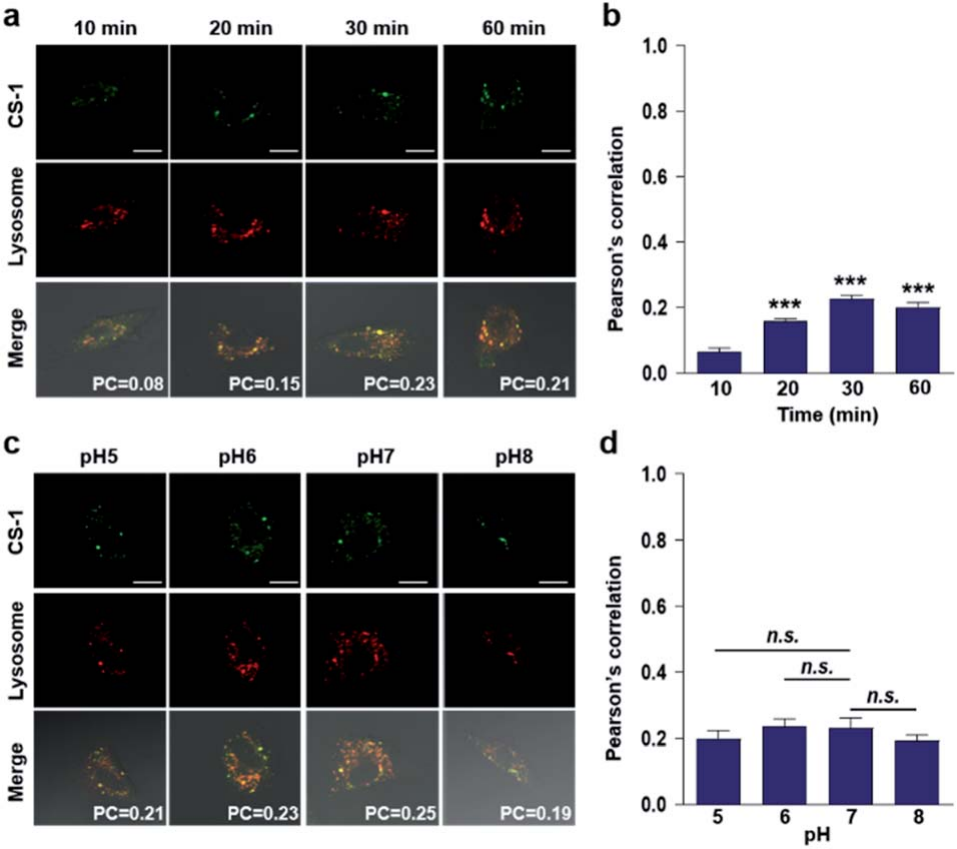
Colocalization of **CS-1** and LysoTracker® staining in normal MRC-5 cells. (a and b) MRC-5 cells, incubated at ∼pH 7.4, were treated with **CS-1** (10 μM) for 10, 20, 30 min and 1 h, together with LysoTracker® Red DND-99 (100 nM) for 30 min. (a) Representative CLSM images for **CS-1** and LysoTracker®. (b) Pearson's correlation coefficients (PC) between **CS-1** and the LysoTracker® as a function of time. (c and d) MRC-5 cells were treated with **CS-1** (10 μM) and LysoTracker® for 30 min at pH 5, 6, 7, and 8. (c) Representative CLSM images for **CS-1** and LysoTracker®, and (d) corresponding PCs at different pH; study replicated in 5 independent experiments. Scale bar: 20 μm. Graphs show mean ± SD. ***P < 0.001 (two-tailed unpaired Mann–Whitney) compared to 10 min group.

In conjunction with lysosomal tracker, time- and pH-dependent fluorescence images with **CS-1** in A549 cells were acquired. For cells incubated in PBS at pH 7.4, the extent of co-localization increased with time from 10–30 min (PC = 0.40 ± 0.05 to 0.74 ± 0.02) (Fig. 3a and b). This time-dependent finding was taken as an indication that **CS-1** moves slowly from the cell surface to the lysosomes as a function of time. As above, the highest colocalization coefficient (mean PC = 0.84 ± 0.04, Fig. 3b) was obtained at pH 5 (Fig. 3c and d). Such a finding is consistent with the assumption that the pH of the lysosomes falls within the pH 4–6 range. In normal MRC-5 cells, the co-localization coefficient in lysosome also increased with time and reached 0.23 ± 0.01 at 30 min; however, the value was still very low compared to what was seen in cancer cells (Fig. 4a and b). Moreover, there was no significant change in the co-localization coefficient over the tested pH 5–8 range (PC = 0.21 ± 0.02) (Fig. 4c and d).

**Fig. 5:**
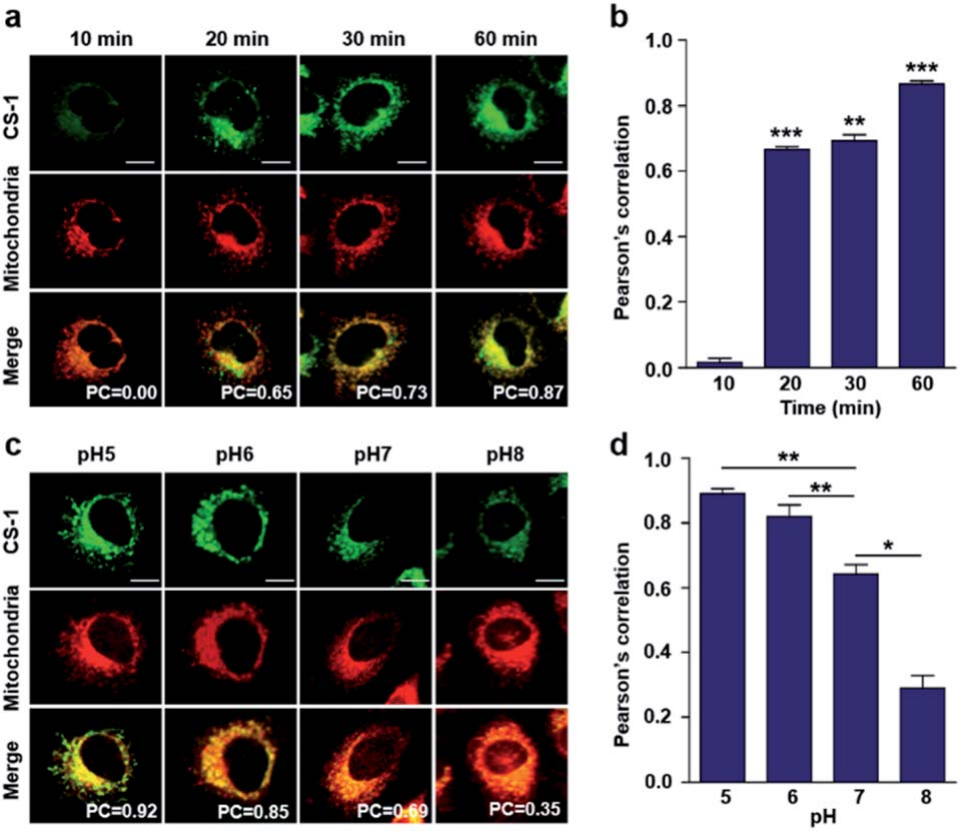
Time- and pH-dependent colocalization of **CS-1** and mitochondria in A549 cancer cells. (a and b) A549 cells were treated with **CS-1** (10 μM) at pH 7.4 for 10, 20, 30 min and 1 h, and subject to MitoTracker® staining (100 nM) for 30 min. (a) Representative CLSM images for **CS-1** and MitoTracker®, and (b) corresponding Pearson's correlation coefficients between **CS-1** and the MitoTracker® plotted as a function of time. (c and d) A549 cells were treated with **CS-1** (10 μM) and MitoTracker® for 30 min at pH 5, 6, 7, and 8. (c) Representative CLSM images for **CS-1** and MitoTracker® and (d) corresponding PCs. Study replicated in 3 independent experiments. Scale bar: 20 μm. Graphs show mean ± SD. *P < 0.05, **P < 0.01, and ***P < 0.001 (two-tailed unpaired Mann–Whitney).

As noted above, rapid oxidative phosphorylation is associated with acidosis during ATP synthesis in cancer-cell mitochondria.^4–6^ We were thus keen to explore whether probe **CS-1** would localize in the mitochondria and could be used to estimate the extent of mitochondrial acidosis, as well as the relative proton concentrations within or near the outer mitochondrial membrane. To test this possibility, probe **CS-1** and MitoTracker® were subject to a time-dependent colocalization study analogous to those described above (*i.e.*, cells incubated in PBS at pH 7.4). We observed that at 60 min post-incubation, probe **CS-1** is largely localized within the mitochondria, as inferred from the fact that the colocalization coefficient with MitoTracker® was maximal at this time (PC = 0.87 ± 0.02) (Fig. 5a and b). On the basis of pH-dependent colocalization studies, we conclude that the extent of colocalization with MitoTracker® is relatively high at pH ≤ 6. Specifically, the overlap coefficient at 60 min is similar to the coefficient seen at pH 5 in the calibration studies (PC = 0.89 ± 0.03; Fig. 5d). Such a finding is taken as evidence that the apparent mitochondrial pH in cancer cells is ∼5.

When the pH of the cell medium used for these cells was reduced to 5, the colocalization coefficient for the plasma membrane, lysosomes, and mitochondria was found to increase to 0.68 ± 0.06, 0.84 ± 0.04, and 0.89 ± 0.03 (Fig. 2, 3, and 5), respectively. The extent of colocalization of **CS-1** in various organelles also varies depending on the pH of the respective organelles. On this basis we suggest that probe **CS-1** localizes to particular organelles in a time-gated manner and that the extent of localization depends on the effective proton (H^+^) concentration. Previously Urano *et al.*^29^ observed that their small molecular pH probe underwent intracellular localization within 10 min. Whereas a longer time is required for **CS-1**, which likely reflects its amphiphilic character.

To obtain further insight into the form of probe **CS-1** within various cellular sub-environments, we carried out dynamic light scattering (DLS) under both acidic (pH 4.5) and alkaline (pH 7.4) conditions. From these studies, which can provide an indication of effective particle size, we conclude that at acidic pH probe **CS-1** self-assembles (Scheme 1b) to form aggregates that are *ca.* 1.6-fold larger in terms of the hydrodynamic radius than the species observed at physiological pH (420 nm *vs.* 260 nm) (Fig. S37†). Furthermore, upon irradiation with UV-light (*λ*_abs_ = 360 nm) for 30 min, the extent of aggregation remains unchanged, which is taken as evidence for the photo-stability of probe **CS-1** (Fig. S38†).^47^ Scanning electron microscopic (SEM) analyses of **CS-1** provided support for the notion that lamellar self-aggregated sheets are formed at acidic pH (pH 4.5); in contrast, at higher pH (pH 7.4) **CS-1** exists in the form of discrete small granular particles (Fig. S39†). Thus, we conclude that self-assembly of probe **CS-1** at lower pH generates a form that is retained for longer times; consequently, probe **CS-1** may be used to read indirectly the pH of intracellular organelles and to assess in a relative and time-dependent manner the pH of various organelles, including ones associated with intracellular acidosis.

### Tumour model studies and *in vivo* tumour labelling

**Fig. 6:**
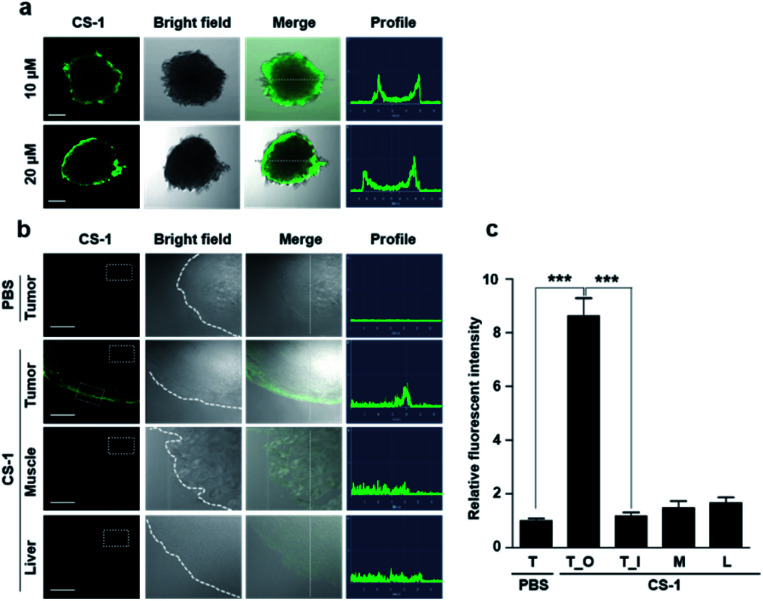
*In vitro* and *ex vivo* fluorescence imaging of cancer cell spheroids and tumour tissue treated with **CS-1**. (a) A549 spheroid cells treated with **CS-1** (10 and 20 μM) for 30 min. Fluorescence images were then captured using a confocal fluorescence microscope. Also shown are the profiles obtained from the images as analysed in the indicated directions. Scale bar: 100 μm. (b and c) A549 xenograft mice were subject to intravenous injection with **CS-1**. Approximately 24 h after treatment with **CS-1** (40 nM), tumour muscle, and liver tissues were collected and frozen in Tissue-Tek optimal cutting temperature (OCT) compound (Sakura, Tokyo, Japan) at −20 °C for 20 min. The frozen tissue samples were sliced to give sheets of ∼5 μm thickness for CLSM imaging. (b) Representative CLSM images for tumour, muscle, and liver tissue slices treated with **CS-1**, compared to the group treated with PBS. The **CS-1** signal was only seen on the edge of the tumour, but not in normal tissues (muscle and liver). Scale bar: 100 μm. (c) Quantitative comparisons of the fluorescence intensities measured in the given ROIs (dotted boxes) positioned in the tumour (T), outer border of the tumour (T_O), inner core region of the tumour (T_I), muscle (M), and liver (L). Graphs show mean ± SD. ***P < 0.001 (two-tailed unpaired Mann–Whitney).

As a predicate to exploring whether **CS-1** might serve as a useful pH probe *in vivo*, we evaluated whether it would label cancer cells with relatively higher fluorescence intensity compared to normal cells under co-culture conditions involving both cancer and normal cells. In a co-culture of A549 cancer and MRC-5 normal cells incubated with probe **CS-1** (10 μM) for 10 min, the relative fluorescence intensity in A549 cancer cells was significantly enhanced (*p* = 0.00345) compared to MRC-5 normal cells (Fig. S40†). Also, we evaluated its ability to label cancer cell spheroids. It was found that A549 spheroids having diameters ∼300 μm could be labelled to produce a green fluorescence response in a dose-dependent manner (Fig. 6a). A relatively high level of emission intensity was seen at the boundary of the cancer spheroids. This finding, which provides support for the core hypothesis that **CS-1** is a useful pH sensitive probe that can label tumour models, is ascribed to the higher proton concentration in the extracellular plasma region of this tumour model. This relatively low local pH leads **CS-1** to self-organize on the cell surface and thus be selectively retained there.

Finally, we performed tumour labelling using **CS-1** in Balb/c nude mice bearing A549-derived xenografts. For these experiments, probe **CS-1** was administered *via* tail vein injection. The mice were sacrificed 24 h post-injection, and the tumours were dissected. As shown in Fig. 6b, slices of tumour tissue produced a strong fluorescence response, particularly in the tumour boundary region. On the other hand, little appreciable response was seen in the muscle and liver sections. As can be seen from an inspection of Fig. 6c, an 8-fold enhancement in the fluorescence intensity was seen at the tumour boundary compared to the tumour core or other organs. This site-specific response is notable in that **CS-1** formally lacks a tumour-targeting ligand other than that provided indirectly as the result of its inherent response to changes in pH.

## Conclusions

The amphiphilic pH probe considered in this study, **CS-1**, was designed to allow for cancer cell and sub-cellular labelling. The fluorescence emission of **CS-1** at 532 nm proved responsive to pH, being roughly 8× more intense at acidic pH as compared to alkaline pH. This led to the consideration that **CS-1** could be used to monitor acidosis in cancer cells. Both FACS and confocal fluorescence imaging data provided support for the notion that A549 and AGS cancer cells could be fluorescently labelled with **CS-1**, whereas normal cells (MRC-5) proved relatively refractory. Based on its fluorescent signature, **CS-1** localizes preferentially in the cell membrane, lysosomes, and mitochondria, and in a time-dependent manner. This finding was taken as evidence that probe **CS-1** can be used to detect pH fluctuations in various cancer cell organelles. A DLS/SEM study led to the suggestion that in acidic environments probe **CS-1** self-assembles to generate amphiphilic structures that localize within various acidic compartments in live cells. Probe **CS-1** was found to label effectively cancer spheroid cells, as well as tumour tissues as inferred from studies involving xenograft-bearing mice. On this basis we suggest **CS-1** may have a role to play in the identification of cancerous cells and tumour tissues and that it could provide a fluorescence-based guide during surgical procedures.

## Experimental section

### Materials, methods and instrumentations

Analytical grade chemicals were obtained commercially and used without further purification unless otherwise indicated. Further details, including the synthesis of compounds **1–7**, **R1**, and **R2**, are provided in the ESI.†

### Synthesis of **CS-1**

Compound **5** (500 mg, 66.67 mmol) was dissolved in dry CH_2_Cl_2_ (5 mL). Trifluoroacetic acid in CH_2_Cl_2_ (20%, 25 mL) was slowly added and the resulting reaction mixture stirred for 3 h at room temperature. The reaction mass was concentrated under high vacuum after the reaction was deemed complete. The mass obtained in this way was dissolved in CH_2_Cl_2_ and then washed with water (2 × 20 mL) and brine (1 × 20 mL). The combined organic layer was dried over anhydrous MgSO_4_ and concentrated under reduced pressure to give **CS-1** (350 mg, 82%). ^1^H NMR (400 MHz, DMSO-d_6_): *δ* 9.40 (s, 2H), 8.61 (d, *J* = 6.8 Hz, 1H), 8.46 (d, *J* = 5.6 Hz, 1H),8.31 (d, *J* = 6.8 Hz, 1H), 7.76 (t, *J* = 6 Hz, 2H), 6.93 (d, *J* = 6.8 Hz, 1H), 4.12 (t, *J* = 5.2 Hz, 2H), 3.81 (d, *J* = 4.0 Hz, 2H), 3.16 (s, 4H), 2.93 (q, *J* = 10.8 Hz, 2H), 2.50 (d, *J* = 0.8 Hz, 2H), 1.58 (s, 4H), 1.23–1.16 (m, 24H), 0.84 (t, *J* = 5.2 Hz, 6H). ^13^C NMR (100 MHz, DMSO-d6): *δ* 172.54, 163.25, 159.03, 149.74, 134.15, 130.99, 129.50, 128.43, 124.85, 122.20, 120.59, 118.47, 116.10, 109.23, 104.36, 54.77, 52.78, 51.80, 49.51, 37.77, 31.24, 28.55, 26.04, 23.11, 22.53, 14.00 ppm. ESI-MS *m*/*z* (M + H): calcd 639.4043, found 639.4122.

### Spectroscopic studies

All reagents and solvents used for spectroscopic studies were received from commercial suppliers and used without further purification. Absorption spectra were recorded on an UV-1800 spectrophotometer (Shimadzu, Japan), and fluorescence spectra were recorded using a fluorescence spectrofluorometer (RF-6000; Shimadzu). Unless otherwise indicated, 3.0 mL samples were used and studied using standard 1 cm quartz cells. A stock solution of **CS-1** was prepared in PBS buffer containing 0.2% DMSO. Excitation and emission were effected at 440 and 531 nm, respectively, with the excitation and emission slit widths both being set at 5 nm. Fluorescence quantum yields were determined using a standard procedure^20^ as described in the ESIa.†

### Cell culture studies

MRC-5 (human lung fibroblast), A549 (human lung adenocarcinoma), and AGS (human gastric cancer) cell lines were purchased from the American Type Culture Collection (ATCC, Rockville, MD, USA), and cultured in a conditioned environment at 5% CO_2_ and 95% humidity at 37 °C. MRC-5 cells were cultured in 1× EMEM, while A549 and AGS cells were cultured in 1× DMEM. In a co-culture of A549 cancer and MRC-5 normal cells, each set of cells were established at a density of 2 × 10^3^ cells in the same μ-slide 8-well plate (Ibidi, Martinsried, Germany), and kept at 37 °C in a humidified incubator for 3 h. The next day, the co-cultured cells were incubated with 10 μM **CS-1** for 10 min. Fluorescence images were acquired on a confocal laser scanning fluorescence microscope (LSM710, Carl Zeiss) equipped with a FITC filter set. Images were analyzed using the ZEN2009 software (Carl Zeiss). All media were supplemented with 10% FBS and 1% penicillin-streptomycin (17-745E; Lonza, USA).

### Fluorescence imaging analyses

MRC-5, A549, and AGS cells were treated with the pH probe, **CS-1**. The working solution was prepared to a concentration of 20 μM in phosphate-buffered saline (PBS) and incubated at 37 °C for 30 min. Cells were washed with PBS and fixed with 4% paraformaldehyde (sc-281692; Santa Cruz Biotechnology, USA) for 10 min before fluorescent imaging. For the **CS-1** and lysosome co-staining study, the cells were incubated with **CS-1** (20 μM) and LysoTracker® (100 nM, LysoTracker® Red DND-99 (L7528), Molecular Probes, Eugene, OR, USA) at 37 °C for 30 min. For the **CS-1** and mitochondria co-staining study, the cells were incubated with a solution consisting of **CS-1** (20 μM) and MitoTracker® (250 nM, MitoTracker® Red CMXRos (L7512), Molecular Probes) at 37 °C for 30 min. To achieve membrane staining, cells were incubated with a solution of **CS-1** (20 μM) and CellMaskTM Deep Red Plasma Membrane stain (250 nM, C10046, Molecular Probes) at 37 °C for 30 min prior to imaging. For the **CS-1** and ER-Tracker® (E34250; Molecular Probes) co-stain studies, a solution of **CS-1** (20 μM) and ER-Tracker® in cell culture medium was used with the test cell culture held at 37 °C for 30 min.

### *In vitro* pH calibration studies

A set of buffer solutions with pH values of 5, 6, 7 and 8.0 and containing calcium gluconate (2 mM), MgCl_2_ (1 mM), nigericin (10 μM), and MOPS (30 mM) was prepared. The pH was adjusted as needed with 1 M KOH or 1 M HCl at room temperature. The cells subject to study were incubated with buffer and **CS-1** for 30 min. The fluorescence was then measured by confocal fluorescence microscopy. All fluorescence microscopic studies were carried out using a confocal laser-scanning microscope (CLSM; LSM710; Zeiss, Jena, Germany). The images were captured using the ZEN 2009 software (version 5.5 SP1; Zeiss). Each experiment was carried out in triplicate with the findings expressed as the average with the associated standard deviation.

### Flow cytometry analyses

MRC-5, A549, and AGS cells pretreated with **CS-1** were analyzed by flow cytometry. The solutions subject to analysis were prepared at concentrations of 10 and 20 μM, respectively, in PBS and incubated at 37 °C for 30 min. Cells were washed with PBS and fixed with 4% paraformaldehyde for 10 min. The cells were then washed in PBS, suspended in FACS buffer, and analyzed immediately using a flow cytometer (FACS CantoII; Becton Dickinson, San Jose, CA, USA). Flow cytometry data from 10 000 cells were collected and analyzed using the FlowJo software (Tree Star, Ashland, USA).

### Tumour spheroids imaging

A549 cells were seeded at a density of 104 cells in each well of poly-HEMA-coated 96-well plates. The plates were centrifuged at 200 g for 10 min and then incubated in a humidified 5% CO_2_ atmosphere at 37 °C. Using this approach single spheroids in each well were obtained whose size varied by less than 10%. After 3 days, the spheroid media was changed, and the spheroids were then incubated in a humidified atmosphere of 5% CO2 at 37 °C for 3–6 days. The spheroids obtained in this way were incubated with **CS-1** at 37 °C for 30 min and then examined using a CLSM system (LSM710; Zeiss).

### *In vivo* studies

A mouse tumour model was established by subcutaneous injection of A549 (2 × 10^6^ cells per mouse) into 6 week male mice (Balb/c nude, *n* = 3 for each group). Two weeks after the initial tumour inoculation, **CS-1** (40 nmol) dissolved in 2.5% DMSO (100 μL) was intravenously administrated *via* the tail vein. Animals were sacrificed at 24 h. The tissue samples (either tumour or normal tissues) were fixed in 4% paraformaldehyde and processed for paraffin embedding using standard procedures. The paraffin embedded tissue sections were cut into slices of 5 μm thickness using a rotary microtome (RM2255; Leica, Wetzlar, Germany) and then mounted on glass slides. The sections were imaged using a confocal laser-scanning microscope (LSM710; Zeiss). All animal experiments were carried out in accord with protocols approved by the Institutional Animal Care and Use Committee (KBSI-AEC1912).

## Conflicts of interest

There are no conflicts to declare.

## Supplementary Material

SC-011-D0SC03795H-s001
