# Supplementary material for: Self-assembled amphiphilic fluorescent probe: detecting pH-fluctuations within cancer cells and tumour tissues
Source: Chem Sci. 2020 Aug 28;11(36):9875–83. doi: 10.1039/d0sc03795h (PMC8162098; doi:10.1039/d0sc03795h)
Supplement: SC-011-D0SC03795H-s001 [file SC-011-D0SC03795H-s001.pdf]

Electronic Supplementary Material (ESI) for Chemical Science.
This journal is © The Royal Society of Chemistry 2020

Supplementary Information

Self-assembled amphiphilic fluorescent probe: Detecting pH-fluctuations within cancer cells and tumor tissues

Soo Yeon Kim,[a,‡] Arup Podder,[b,‡] Hyunseung Lee,[a] Youn-Joo Cho,[a,c] Eun Hee Han,[a] Sabina Khatun,[b] Jonathan L. Sessler,[d,]* Kwan Soo Hong,[a,c,]* and Sankarprasad Bhuniya[b,e]*

[a]Research Center for Bioconvergence Analysis, Korea Basic Science Institute, Cheongju 28119, Korea

[b]Amrita Centre for Industrial Research & Innovation, Amrita Vishwa Vidyapeetham, Ettimadai, Coimbatore 641-112, India

[c]Graduate School of Analytical Science and Technology, Chungnam National University, Daejeon 34134, Korea

[d]Department of Chemistry, University of Texas at Austin, Austin, Texas 78712-1224, USA

[e]Centre for Interdisciplinary Science, JIS Institute of Advanced Studies and Research, JIS University, Kolkata 700-091, India.

[‡]These authors contributed equally to this work

*To whom correspondence should be addressed: sessler@cm.utexas.edu (J.L. Sessler), kshong@kbsi.re.kr (KS Hong), b_sankarprasad@cb.amrita.edu (S. Bhuniya).

Table of contents

Materials, methods, and instrumentation ……………………………………………………………………………………….. S3

Reaction scheme and mechanism for proton-based turn on fluorescent response ………………………………. S4

Reagents and reaction conditions …………………………………………………………………………………………….. S4

Synthesis of 1-7 **…….. S5**

Synthesis of CS-1 **……….. S7**

Synthesis of R1 and R2 **……… S8**

$^{1}H/^{13}C$ NMR and ESI-MS spectra of compounds 1-6 ………………………………………………………………………. S9

$^{1}H/^{13}C$ NMR and ESI-MS spectra of compound CS-1 ……………………………………………………………………… S18

$^{1}H/^{13}C$ NMR and ESI-MS spectra of compounds R1 and R2 …………………………………………………………… S19

UV absorption & fluorescence spectrum of CS-1 at varying pH……………………………………………………… S23

Quantum yields and Fluorescent emission of CS-1 in various analytes, and at pH 4.0/8.5…………………… S24

Plot of pH *vs* log[(I_{max}-I)(I-I_{min})] ………………………………………………………………………………………… S25

Changes in the fluorescence spectra of R1 and R2 ……………………………………………………………………… S25

In vitro cell cytotoxicity of CS-1 ……………………………………………………………………………………………… S26

Fluorescence imaging of cancer and normal cells treated with CS-1 …………………………………………… S27

Fluorescence ascribed to CS-1 in various organelles of MRC-5 normal and A549 cancer cells …………… S28

Dynamic light scattering (DLS) and SEM analyses of CS-1 at pH 4.5 and pH 7.4 ……………………………… S29

SEM images of CS-1 at pH 4.5 and 7.4 ……………………………………………………………………………………… S31

Fluorescence imaging in co-cultured MRC-5 normal and A549 cancer cells treated with CS-1. …………. S32

Supplementary references ………………………………………………………………………………………………………. S33

Materials, methods, and instrumentation

N-(2-Bromoethyl)phthalimide (Sigma), dioctylamine (Sigma), hydrazine hydrate (Sigma), ethylenediamine (Sigma), *tert*-butyl bromoacetate (TCI), n-butylamine (Sigma), 2-ethanol amine (Sigma), ethoxy ethanol (TCI), K_2CO_3 (Rankem), EtOH (Mark), TFA (Sigma-Aldrich), acetonitrile (Avra), dichloromethane (Sigma) were purchased commercially and used without further purification. Flash column chromatography was performed using silica gel (100-200 mesh) and analytical thin layer chromatography was performed using silica gel 60 (pre-coated 0.25 mm sheets). Mass spectra were recorded on an Ion Spec HiRes ESI mass spectrometer (SESI-MS, Fisher Scientific, USA). NMR spectra were collected on a 400 MHz spectrometer (Bruker, Germany).

Reaction scheme and proposed mechanism for proton-based turn on fluorescent response:

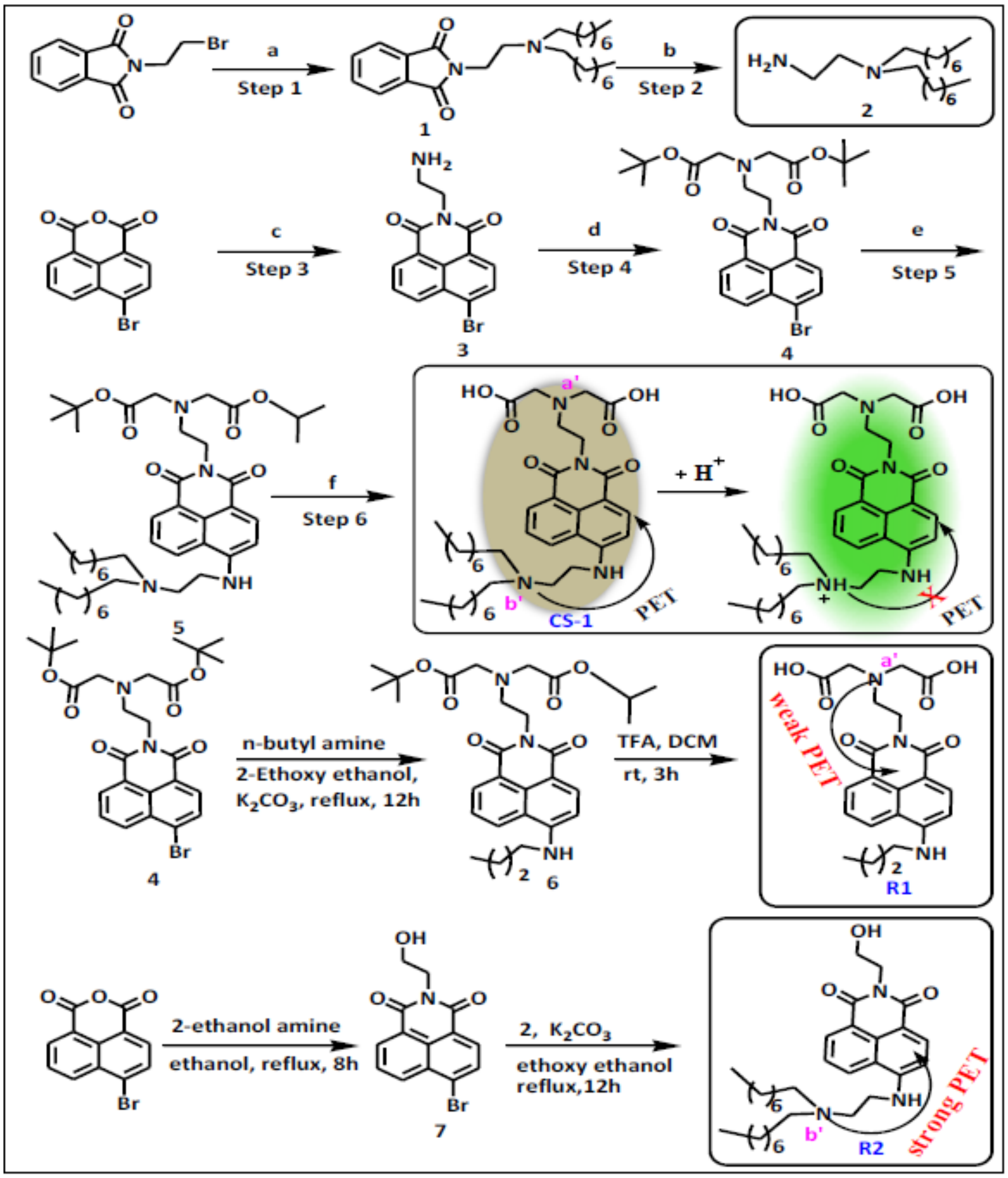

Reagents and reaction conditions: (a) n-dioctylamine, K_2CO_3, ACN, 70°C, 12 h. (b) NH_2NH_2,H_2O, ACN, RT, 12 h. (c) ethylenediamine, EtOH, RT, 2 h. (d) *tert*-butyl bromoacetate, K_2CO_3, acetonitrile (ACN), 70°C, 12 h. (e) Compound **2**, ethoxyethanol, reflux, 12 h. (f) TFA, dichloromethane (DCM), RT, 3 h.

Synthesis of 1: To a stirred solution of N-(2-bromoethyl)phthalimide (6 g, 23.72 mmol) in acetonitrile n-dioctylamine (8.6 g, 35.58 mmol) and K_2CO_3 (13.1 g, 94.88 mmol) were added. The reaction mixture was stirred for 12 h at 70°C until deemed complete by TLC analysis. It was then allowed to cool to room temperature before being diluted with water (50 mL) and then extracted with ethyl acetate (3 × 50 mL). The combined organic layer was washed with brine, then dried over anhydrous $MgSO_4$ and concentrated under reduced pressure to obtain a crude form of the product. This crude material was passed through a silica gel column using ethyl acetate (10%) in hexanes as the eluent. The fractions containing the desired compound were collected and taken to dryness under reduced pressure (rotary evaporator followed by drying in vacuum) to afford compound **1** (6.2 g, 63.36%) as a pale brown liquid. ^1H NMR (400 MHz, $CDCl_3$): δ 7.77 (q, *J* = 5.28 Hz, 2H), 7.63(q, *J* = 5.48 Hz, 2H), 3.68 (t, *J* = 6.76 Hz, 2H), 2.63 (t, *J* = 6.8 Hz, 2H,), 2.37 (t, *J* = 7.28 Hz, 4H), 1.28 (m, 24H), 0.81 (t, *J* = 6.8 Hz, 6H). ^{13}C NMR (100 MHz, $CDCl_3$): δ 168.38, 133.73, 132.26, 123.06, 54.22, 51.42, 36.19, 31.85, 29.60, 27.47, 22.66, 14.10. ESI-MS m/z $[M+H]^+$: calcd. 415.3246, found 415.3225.

Synthesis of 2: The phthalimide protected compound **1** (6 g, 14.49 mmol) was dissolved in acetonitrile. Hydrazine hydrate (2.90 g, 57.96 mmol) was then added slowly and the resulting reaction mixture was stirred for 4 h at room temperature. The volatiles were removed under reduced pressure and water was added. The aqueous phase was extracted three times with chloroform and the combined organic layer was dried with anhydrous $MgSO_4$. Further evaporation under reduced pressure afforded the pure amine product as a red-brown oil (3.8 g, yield-92.2%). ^1H NMR (400 MHz, $CDCl_3$): δ 2.71-2.57 (m, 2H), 2.45-2.34 (m, 6H), 1.39 (s,4H), 1.24 (s, 20H), 0.86 (t, *J* = 4.64 Hz, 6H). ^{13}C NMR (100 MHz, $CDCl_3$): δ 56.80, 54.28, 39.58, 31.84, 29.55, 27.51, 22.63,14.06. ESI-MS m/z $[M+H]^+$: calcd. 285.3191, found 285.3272.

Synthesis of 3: Ethylenediamine (2.4 mL, 36.1 equiv) was added to a stirred solution of 4-bromo-1,8-naphthalic anhydride (2 g, 7.22 mmol) in ethanol. This mixture was then stirred in the dark for 2 h at room temperature. After the reaction was deemed complete on the basis of TLC analysis, the volatiles were removed using a rotary

evaporator. At this point, 30 mL DI-water was added to the flask and the contents extracted with CH_2Cl_2 (3 x 30 mL). After drying with $MgSO_4$, the solution was filtered and the volatiles removed under vacuum. The resulting residue was then purified by flash column chromatography (CH_2Cl_2: MeOH = 90:10). Compound **3** was obtained (1.2 g, yield 52%) as light yellow colored powder. ^{1}H NMR (400 MHz, DMSO-d_6) δ 8.44 (s, 2H), 8.20 (d, *J* = 36 Hz, 2H), 7.90 (s, 1H), 4.05 (s, 2H), 2.84 (s, 2H). ^{13}C NMR (100 MHz, DMSO-d_6) δ 163.49, 132.82, 131.81, 131.19, 130.03, 129.40, 128.60, 123.17, 122.40, 63.39, 42.69. ESI-MS m/z $[M+H]^+$: calcd. 319.0004, found 319.0088.

Synthesis of 4: To a stirred solution of compound **3** (1 g, 3.15 mmol) in acetonitrile, tert-butyl bromoacetate (1.67 mL, 6.3 mmol) and potassium carbonate (1.74 g, 12.6 mmol) were added. The reaction mixture was stirred for 12 h at 70°C. It was then allowed to cool to room temperature before being diluted with water (50 mL) and extracted with ethyl acetate (3 x 50 mL). Then total organic layer was washed with brine, then dried over $MgSO_4$ and concentrated under reduced pressure to obtain the product in crude form. This crude material was purified by column chromatography using ethyl acetate (25%) in hexanes as the eluent. After collecting the appropriate fractions, and drying under reduced pressure (rotary evaporator, high vacuum), compound **4** (1.3 g, yield 73.36%) was obtained in the form of an off-white solid. ^{1}H NMR (400 MHz, DMSO-d_6): δ 8.41 (t, *J* = 3.44 Hz, 2H), 8.19-8.08 (m, 2H), 7.88 (s, 1H), 4.05 (d, *J* = 3.32 Hz, 2H), 3.38 (s, 4H), 2.87 (t, *J* = 6.2 Hz, 2H), 1.27 (s, 18H). ^{13}C NMR (100 MHz, DMSO-d_6): δ 170.49, 163.31, 132.86, 131.84, 131.22, 130.10, 129.35, 128.70, 123.27, 122.50, 80.58, 55.80, 51.23, 28.23, 19.33. ESI-MS m/z $[M+H]^+$: calcd. 547.1365, found 547.1440.

Synthesis of 5: Compound **2** (2.6 g, 9.15 mmol) and Compound **4** (1 g, 1.83 mmol) were dissolved in 2-ethoxy ethanol (15 mL) in a single neck round bottom flux and then stirred at 140°C for 16 h at which point the reaction was deemed complete as judged by TLC analysis (40% ethyl acetate, EA). The reaction mixture was then dissolved in 50 mL water and extracted with EA (3 x 30 mL). The combined organic fractions were dried over $MgSO_4$ and concentrated under reduced pressure to give the product of crude form. This crude material was purified by column chromatography (50% EA in hexanes). Compound **5** was obtained as yellowish-green semiliquid

compound (1.2 g, yield 88%). ^{1}H NMR (400 MHz, $CDCl_3$): δ 8.49 (d, *J* = 7.28 Hz, 1H), 8.35 (d, *J* = 8.36 Hz,1H), 8.01 (d, *J* = 8.32 Hz,1H), 6.59 (d, *J* = 8.44 Hz, 1H), 6.42 (s, 1H), 4.24 (t, *J* = 7.08 Hz, 2H), 3.52 (s, 4H), 3.27 (d, *J* = 4.52 Hz, 2H), 3.02 (t, *J* = 7.36 Hz, 2H), 2.79 (t, *J* = 5.72 Hz, 2h), 2.45 (t, *J* = 7.08 Hz, 4H), 1.36 (s, 18H), 1.19 - 1.12 (m, 24H), 0.79 (t, J = 6 Hz, 6H). ^{13}C NMR (100 MHz, $CDCl_3$): δ 170.92, 164.70, 149.69, 134.56, 131.00, 129.93, 126.14, 124.51, 123.10, 120.52, 109.97, 104.51, 80.54, 69.86, 66.60, 63.99, 55.74, 53.29, 51.64, 31.85, 29.70, 28.15, 27.60, 26.50, 22.63, 19.50, 14.11 ppm. ESI-MS m/z $[M+H]^+$: calcd. 751.5295, found 751.5364.

***Synthesis of 6*:** To a stirred solution of compound **4** (200 mg, 0.37 mmol) in 2-ethoxy ethanol (10 mL) n-butyl amine (180 µL, 1.83 mmol) was added. The resulting reaction mixture was then heated at reflux (140°C) for approximately 16 h. When TLC analysis (30% EA in hexanes) revealed that the reaction was complete, water was added (50 mL) before extracting with EA (3 x 30 mL). The combined organic layer was dried over $MgSO_4$ and concentrated under reduced pressure to give compound **5** in crude form. Following purification by column chromatography (40% EA in hexanes), compound **5** was obtained as yellowish-green semi-liquid (148 mg, yield 75%). ^{1}H NMR (400 MHz, $CDCl_3$): δ 8.37 (d, *J* = 8.44, Hz, 2H), 8.01 (d, *J* = 10.0 Hz, 1H), 7.53 (t, *J* = 8.0 Hz, 1H), 6.59 (d, *J* = 8.04 Hz, 1H), 6.52 (s, 1H), 3.52 (s, 6H), 2.98 (t, *J* = 7.16 Hz, 2H), 2.76 (t, *J* = 5.6 Hz, 2H), 1.53 (s, 2H), 1.36 (s, 20H), 0.98 (t, J = 4.92 Hz, 3H). ^{13}C NMR (100 MHz, $CDCl_3$): δ 170.92, 164.70, 162.52, 134.58, 131.00, 129.93, 126.14, 12309, 120.52, 115.51, 109.97, 80.58, 56.80, 55.78, 51.64, 48.69, 31.85, 28.15, 19.49, 14.12 ppm. ESI-MS m/z $[M+H]^+$: calcd. 540.2995, found 540.3340.

***Synthesis of 7*:** Compound **7** was synthesized according to a reported method.[1]

***Synthesis of CS-1*:** Compound **5** (500 mg, 66.67 mmol) was dissolved in dry dichloromethane (DCM; 5 mL). Then, TFA in DCM (20%, 25 mL) was added slowly and the resulting reaction mixture stirred for 3 h at room temperature. The reaction mixture was then concentrated under reduced pressure (rotary evaporator followed by high vacuum) to give the product in crude form. This crude product was dissolved in DCM, washed with water (2 x 20 mL) and

brine (1 x 20 mL). The combined organic layer was dried over anhydrous $MgSO_4$ and concentrated under reduced pressure to give **CS-1** (350 mg, yield 82%). ^{1}H NMR (400 MHz, DMSO-d_6): δ 9.40 (s, 2H), 8.61 (d, *J* = 6.8 Hz,1H), 8.46 (d, *J* = 5.6 Hz,1H), 8.31 (d, *J* = 6.8 Hz, 1H), 7.76 (t, *J* = 6 Hz, 2H), 6.93 (d, *J* = 6.8 Hz, 1H), 4.12 (t, *J* = 5.2 Hz, 2H), 3.81 (d, *J* = 4.0 Hz, 2H), 3.16 (s, 4H), 2.93 (q, *J* = 10.8 Hz, 2H), 2.50 (d, *J* = 0.8 Hz, 2H), 1.58 (s, 4H), 1.23-1.16 (m, 24H), 0.84 (t, *J* = 5.2 Hz, 6H). ^{13}C NMR (100 MHz, DMSO-d_6): δ 172.54, 163.25, 159.03, 149.74, 134.15, 130.99, 129.50, 128.43, 124.85, 122.20, 120.59, 118.47, 116.10, 109.23, 104.36, 54.77, 52.78, 51.80, 49.51, 37.77, 31.24, 28.55, 26.04, 23.11, 22.53, 14.00 ppm. ESI-MS m/z $[M+H]^+$: calcd. 639.4043, found 639.4122.

Synthesis of R1**:** For compounds **R1** to **R2** we followed same procedure as described above for the synthesis of **CS-1.** Compound **R1** was obtained as a black solid (90 mg, 80.25%). ^{1}H NMR (400 MHz, DMSO-*d*6): δ 9.46 (s, 2H); 8.33 (d, *J* = 6.4 Hz, 2H), 7.73 (d, *J* = 6.8 Hz, 2H), 6.91 (d, *J* = 6.8 Hz, 1H), 4.09 (s, 1H), 3.50 (s, 6H), 3.34 (s, 2H), 2.50 (d, *J* = 17.2 Hz, 2H), 1.56 (s, 2H), 1.23-1.16 (m, 2H), 0.84 (t, *J* = 5.0 Hz, 3H). ^{13}C NMR (100 MHz, DMSO-d6): δ 164.7, 162.88, 157.54, 155.52, 149.22, 129.22, 129.24, 114.24, 11.26, 110.80, 109.90, 81.23, 28.29, 21.78, 8.34. HRMS m/z $[M+H]^+$: calcd. 428.1743, found 428.1823.

Synthesis of R2**:** To prepare **R_2,** compound **7** (200 mg, 0.63 mmol) and compound **2** (888 mg, 3.14 mmol) were combined in 2-ethoxy ethanol (5 mL) and then allowed to react following the same protocol as used for the synthesis for **5.** Compound **R_2** was obtained as yellow solid. ^{1}H NMR (400 MHz, $CDCl_3$): δ 8.56 (d, *J* = 6.96 Hz, 1H); 8.45 (d, *J* = 8.36 Hz,1H), 8.10 (d, *J* = 7.96 Hz, 1H); 7.60 (t, *J* = 7.56 Hz, 1H), 6.65 (t, *J* = 7.6 Hz, 1H), 4.45 (d, *J* = 3.8 Hz, 2H), 3.99 (d, *J* = 4.56 Hz, 2H), 3.35 (s, 2H), 2.80 (t, *J* = 8 Hz, 4H), 2.54 (t, 5.8 Hz, 2H), 1.75 (s, 2H), 1.23 (t, *J* = 7.04 Hz, 24H), 0.86 (t, *J* = 6.56 Hz, 6H). ^{13}C NMR (100 MHz, $CDCl_3$): δ 164.59, 149.08, 134.06, 130.35, 128.91, 125.57, 123.53, 121.59, 119.39, 108.26, 103.58, 61.31, 52.24, 50.59, 47.57, 41.81, 38.94, 30.79, 28.53, 26.59, 21.61, 13.05 ppm. ESI-MS m/z $[M+H]^+$: calcd. 524.3773, found 524.3853.

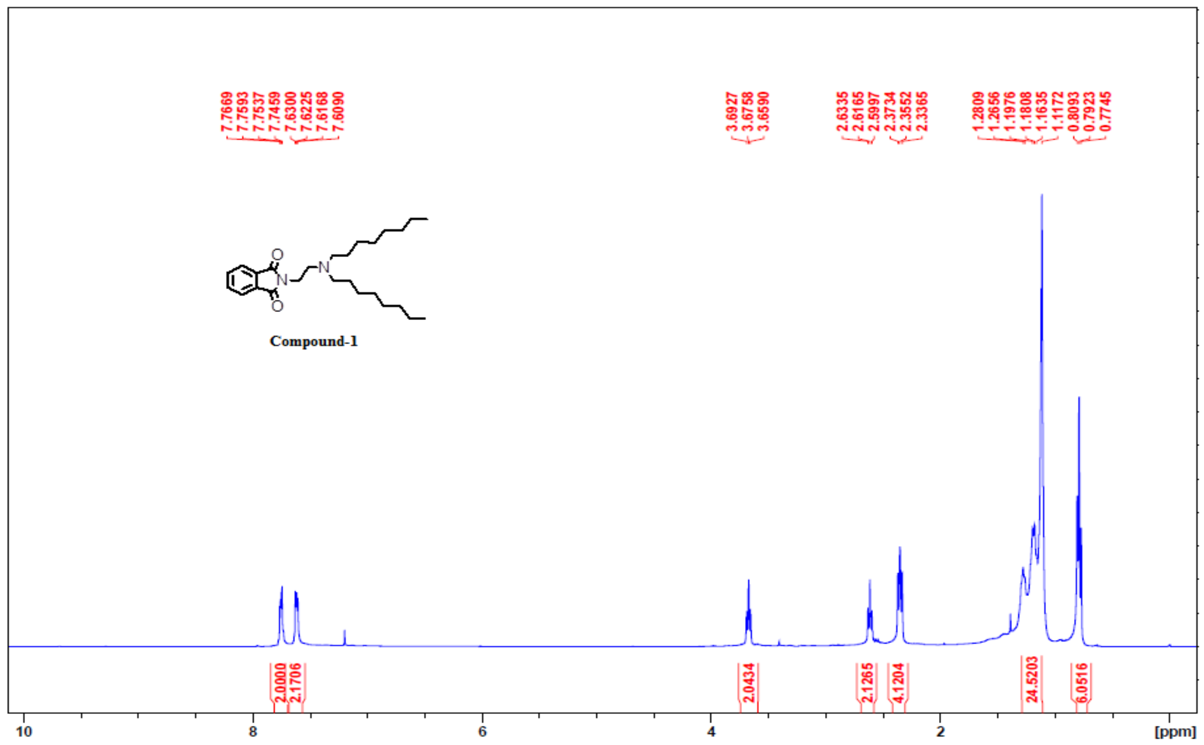

Figure S1. ^{1}H NMR spectrum of compound **1** in $CDCl_3$.

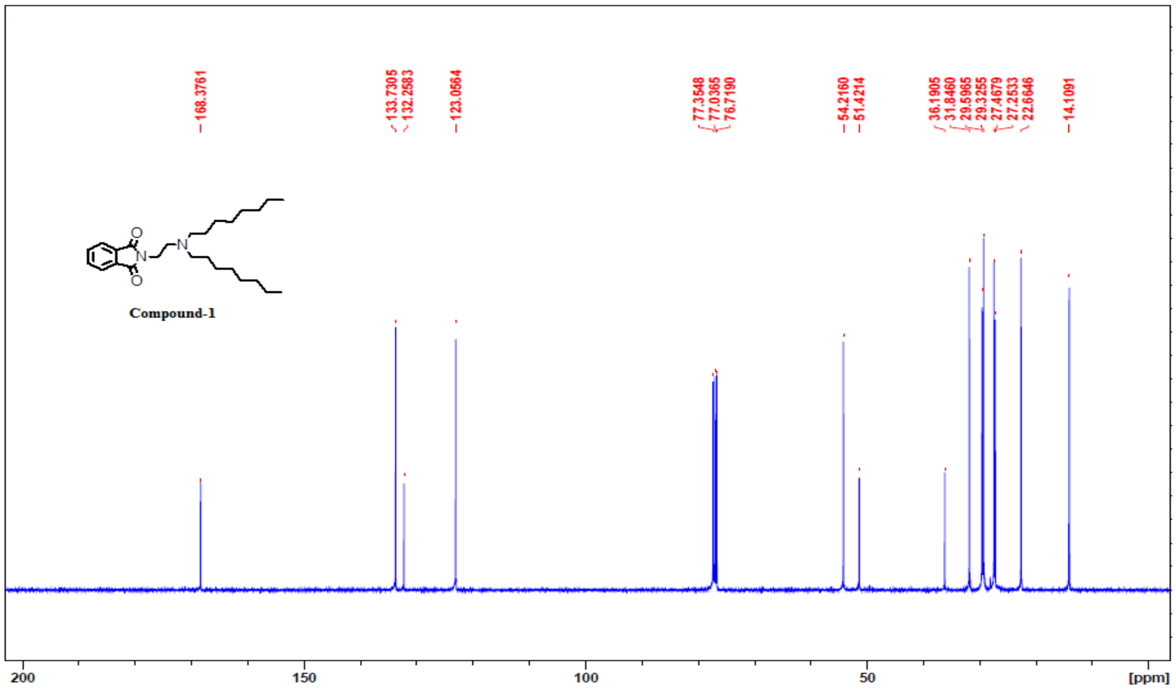

Figure S2. ^{13}C NMR spectrum of compound **1** in $CDCl_3$.

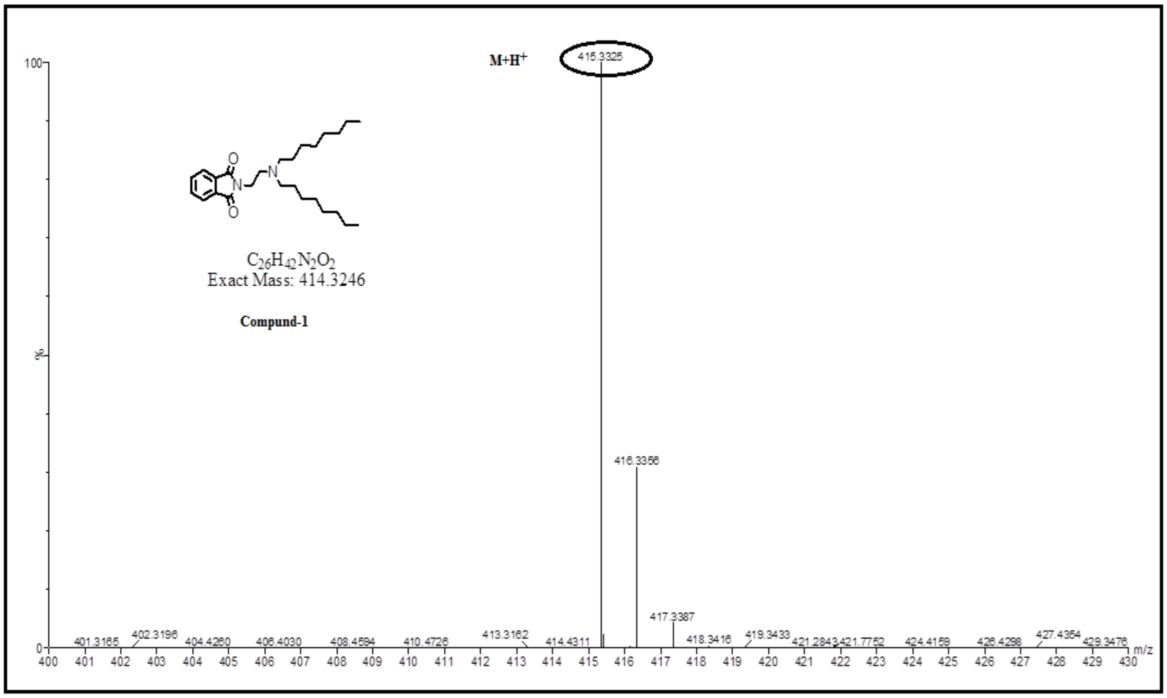

Figure S3. ESI-MS of compound **1**.

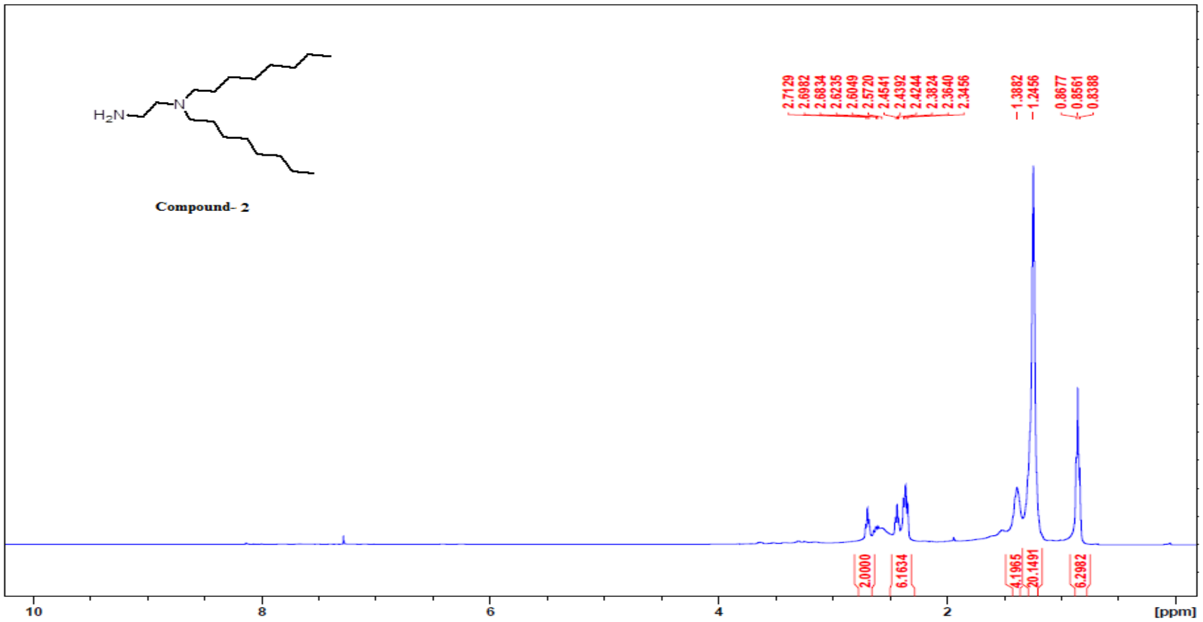

Figure S4. ^{1}H NMR spectrum of compound **2** in $CDCl_3$.

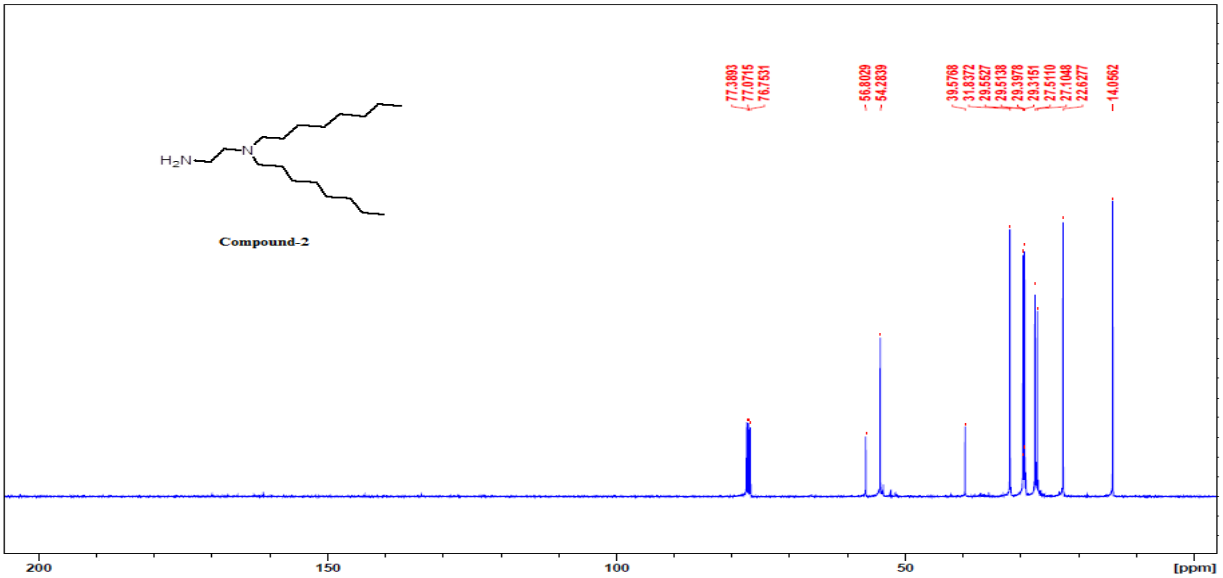

Figure S5. ^{13}C NMR spectrum of compound **2** in $CDCl_3$.

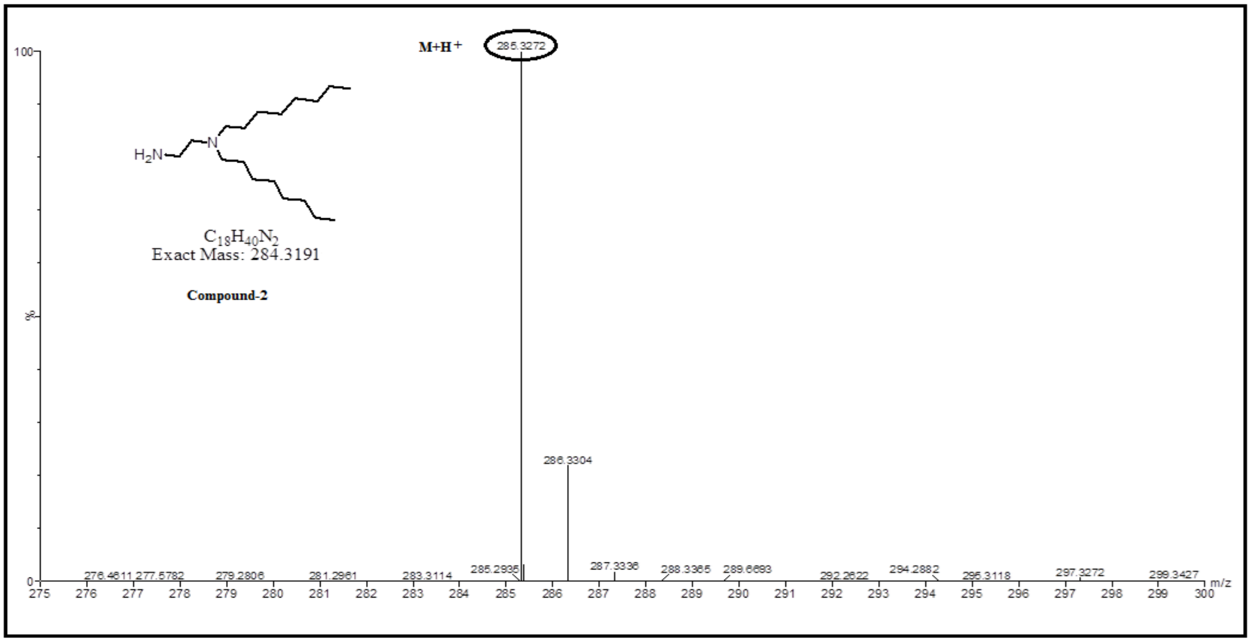

Figure S6. ESI-MS of compound **2**.

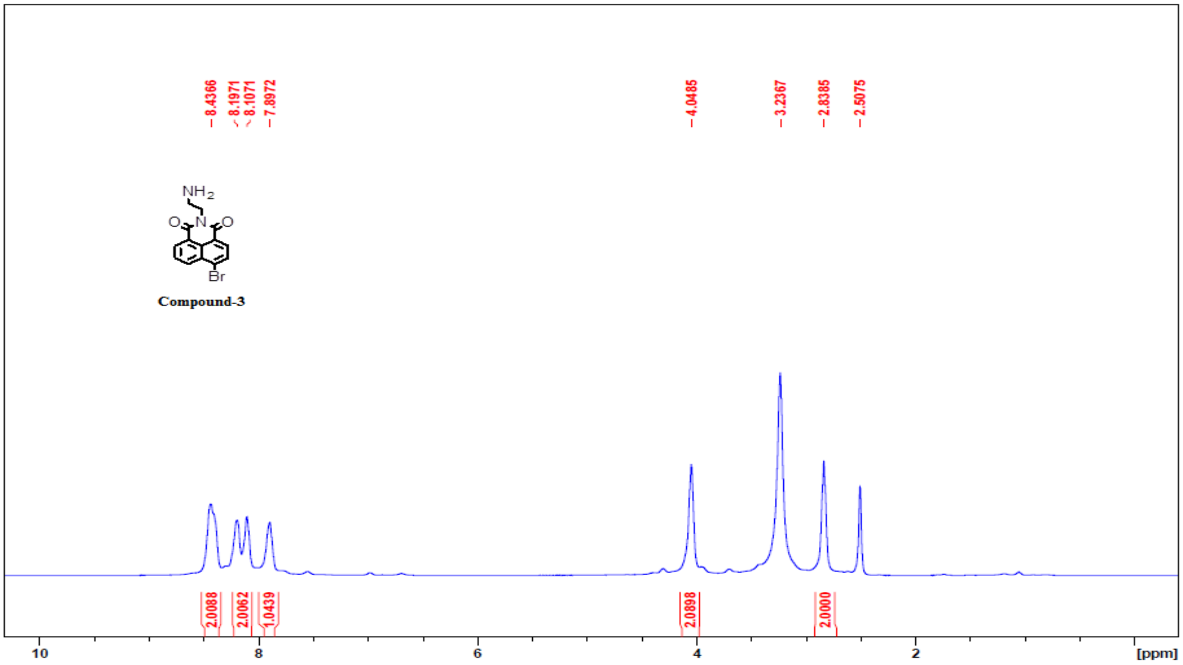

Figure S7. ^{1}H NMR spectrum of compound **3** in DMSO-d_6.

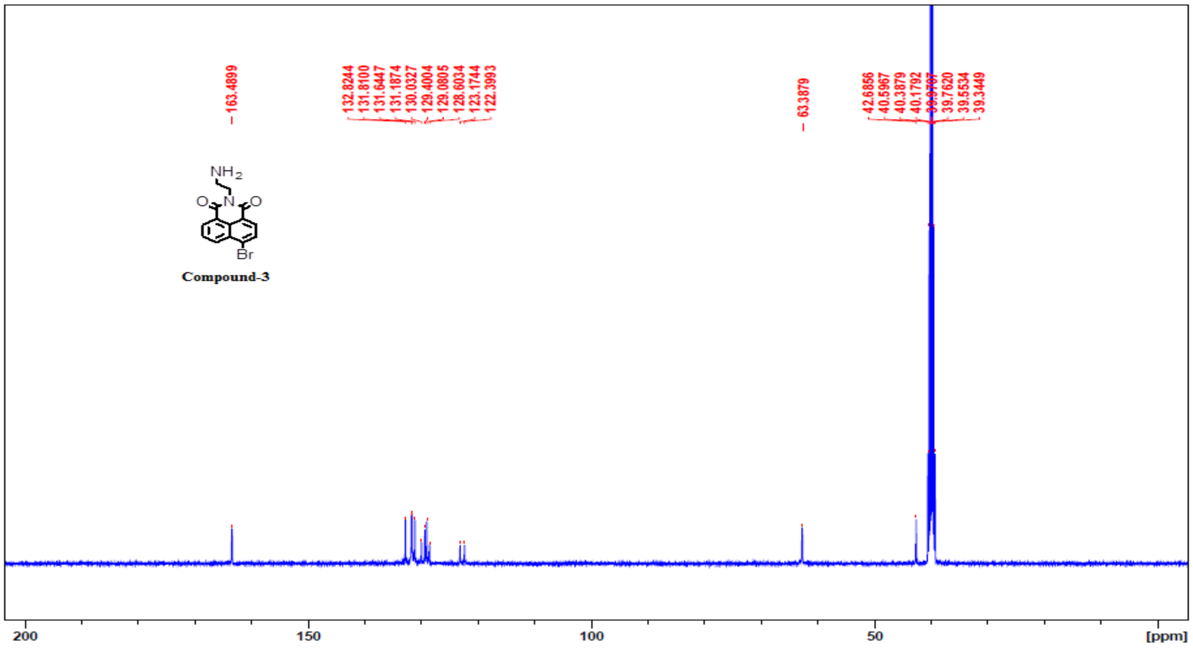

Figure S8. ^{13}C NMR spectrum of compound **3** in DMSO-d_6.

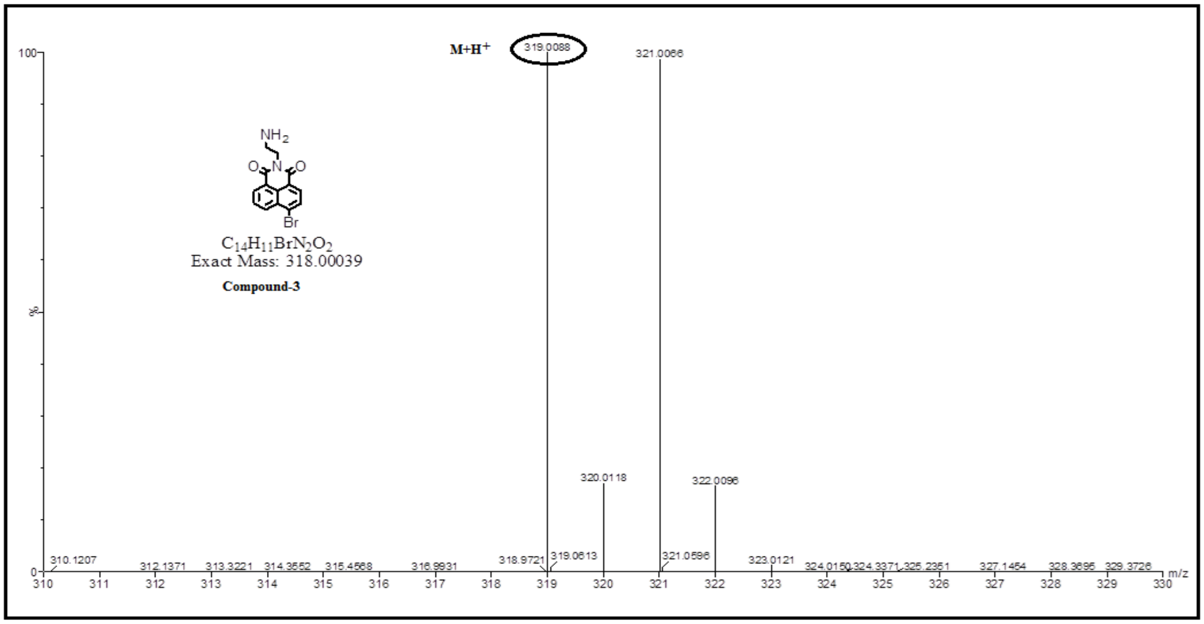

Figure S9. ESI-MS of compound **3**.

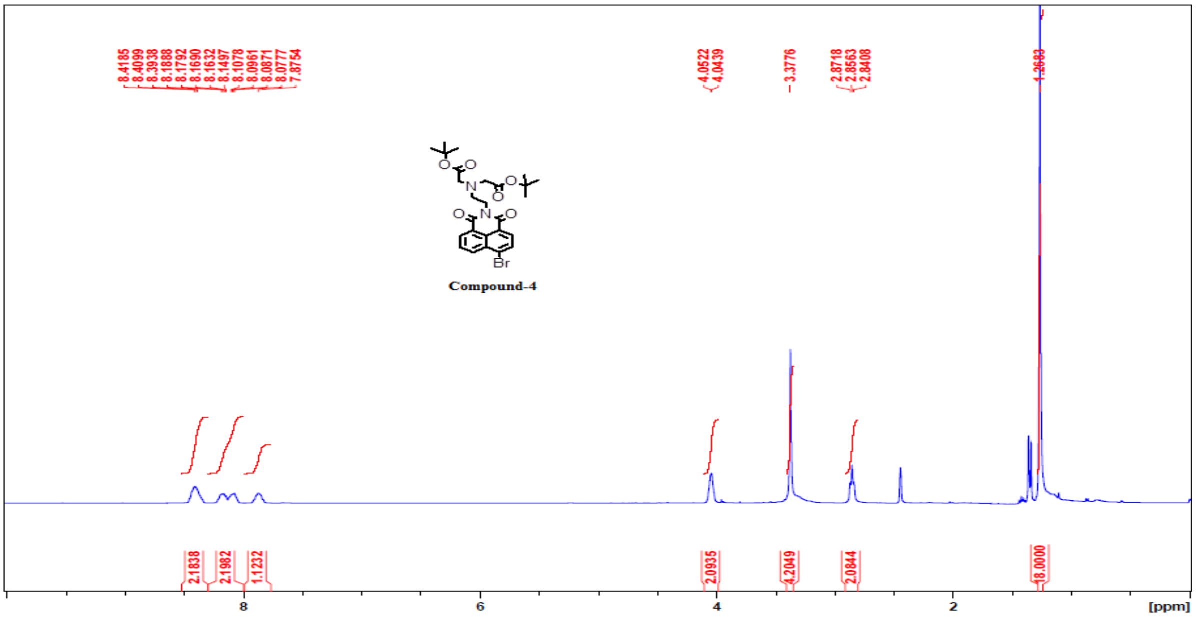

Figure S10. ^{1}H NMR spectrum of compound **4** in DMSO-d_6.

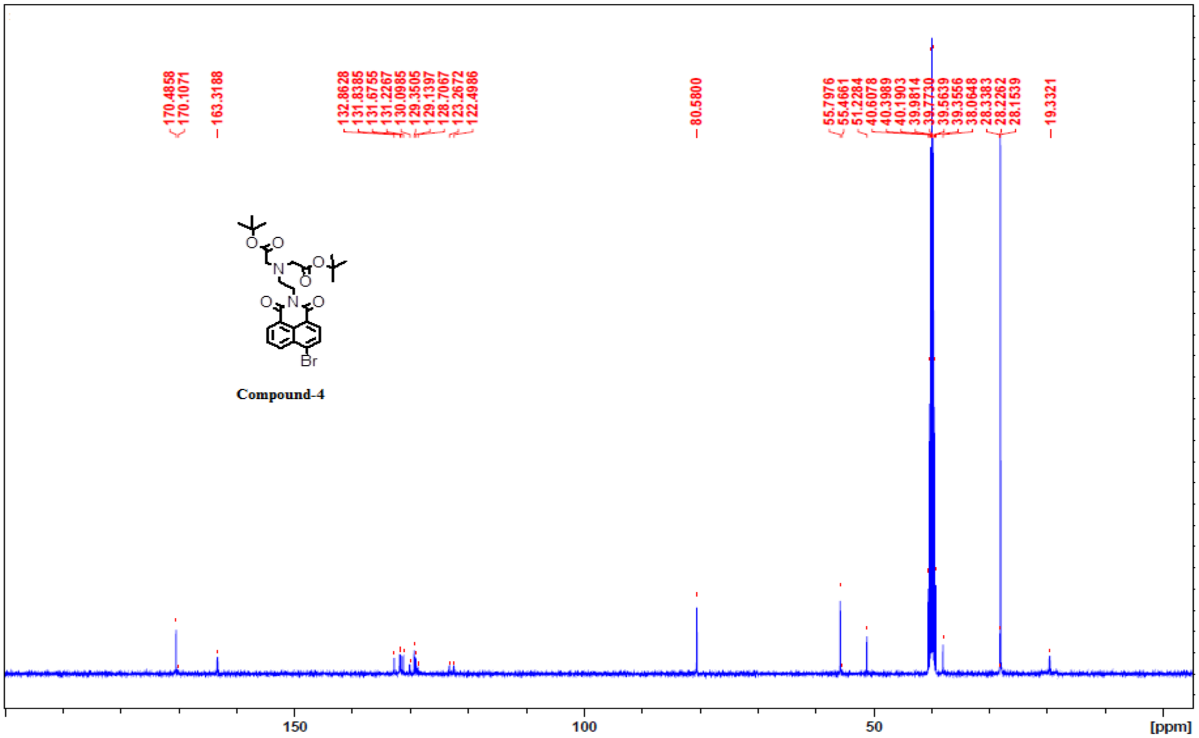

Figure S11. ^{13}C NMR spectrum of compound **4** in DMSO-d_6.

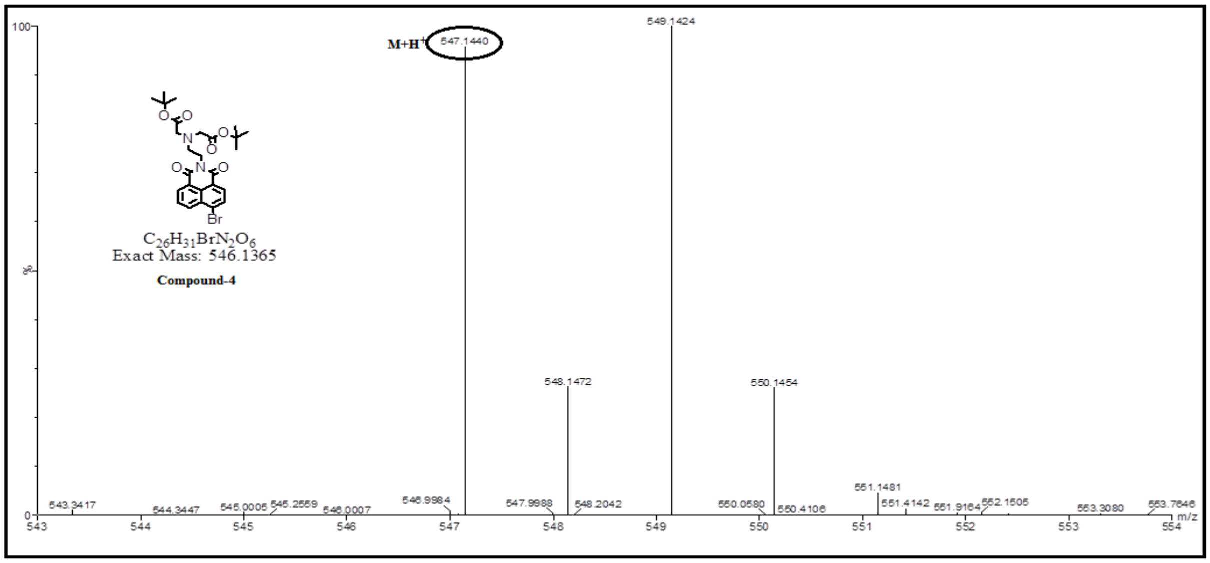

Figure S12. ESI-MS of compound **4**.

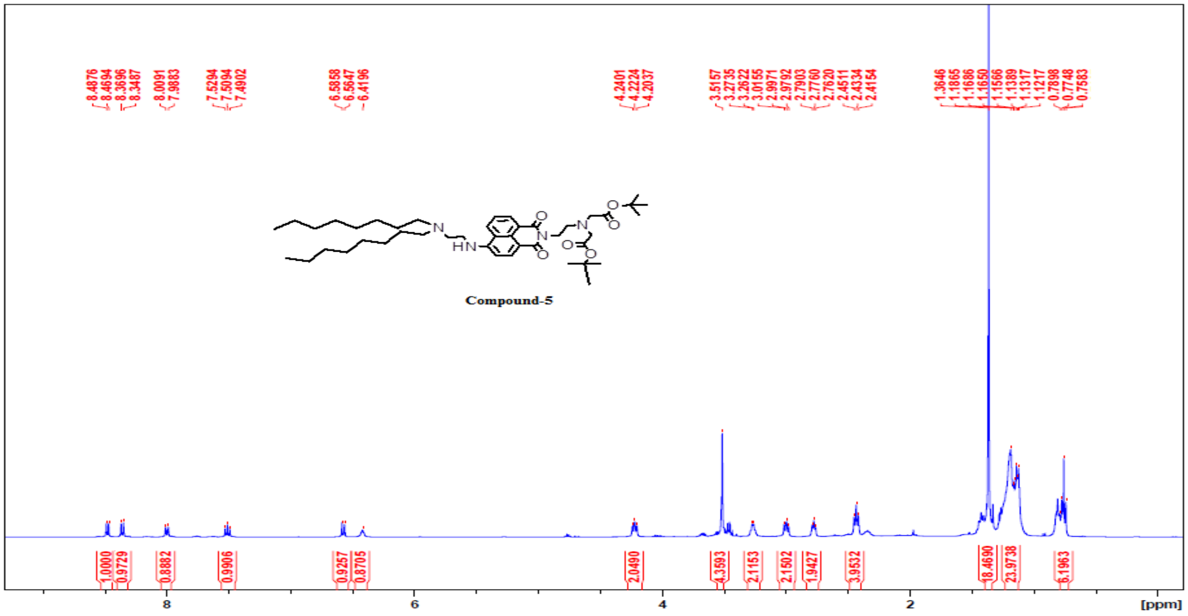

Figure S13. ^{1}H NMR spectrum of compound **5** in $CDCl_3$.

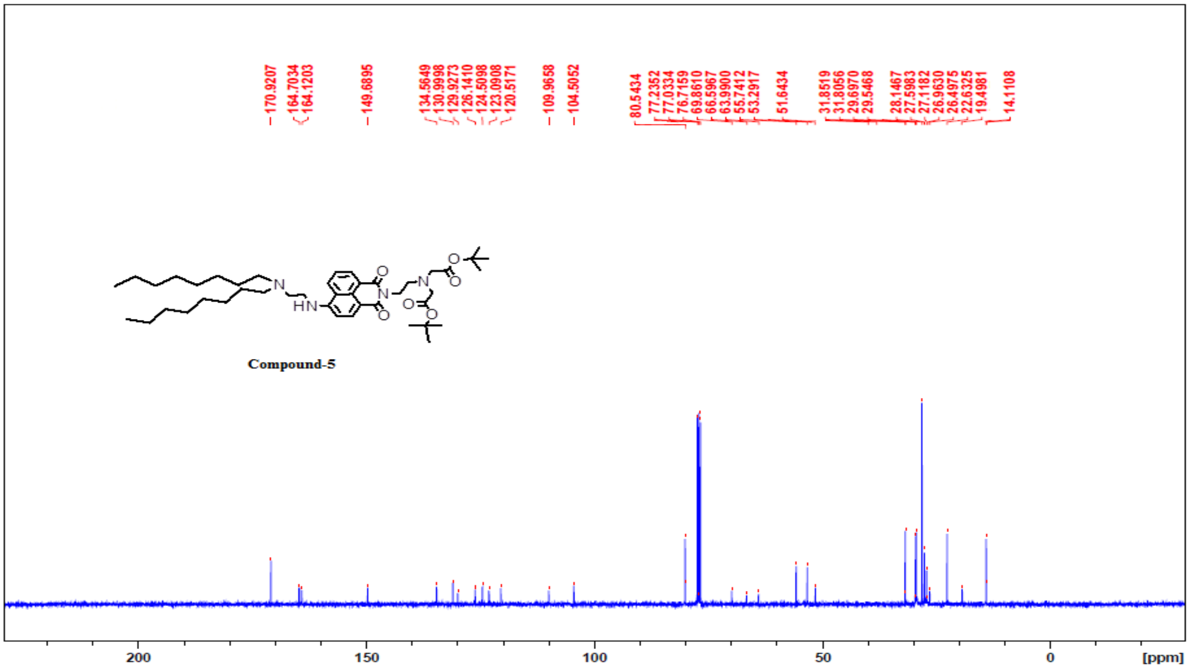

Figure S14. ^{13}C NMR spectrum of compound **5** in $CDCl_3$.

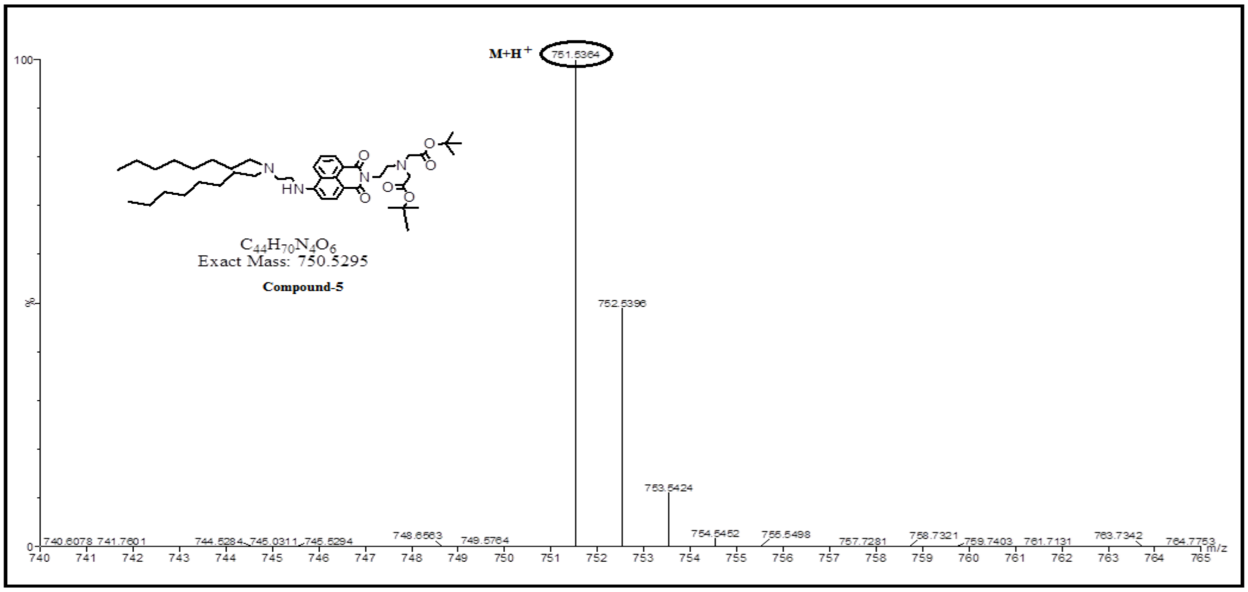

Figure S15. ESI-MS of compound **5**.

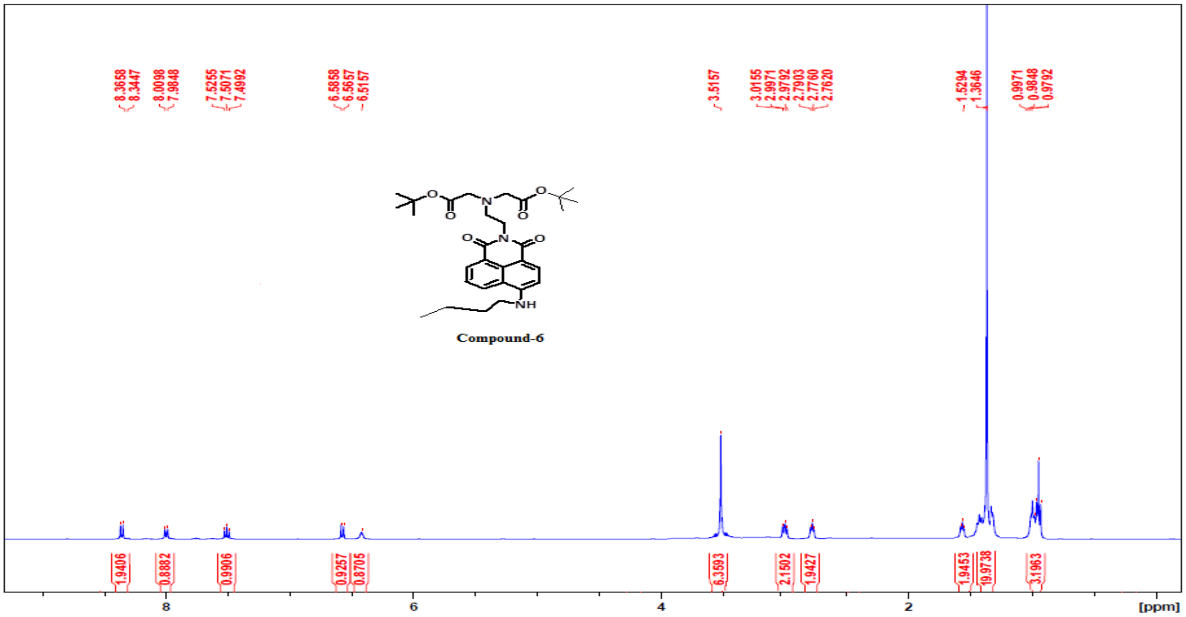

Figure S16. ^{1}H NMR spectrum of compound **6** in $CDCl_3$.

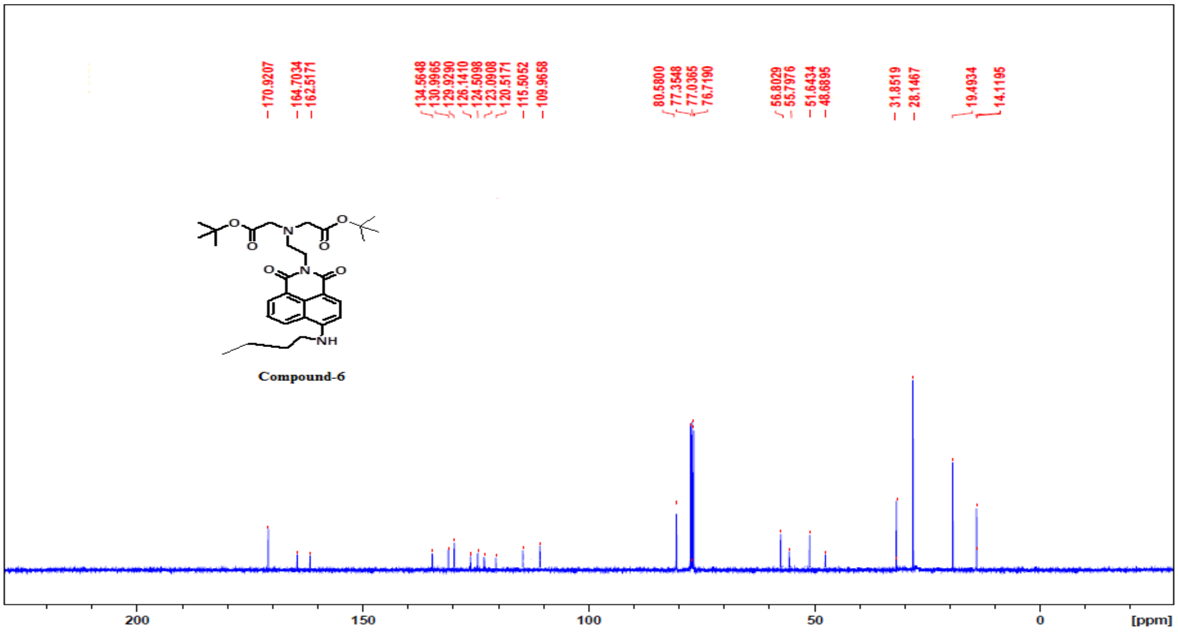

Figure S17. ^{13}C NMR spectrum of compound **6** in $CDCl_3$.

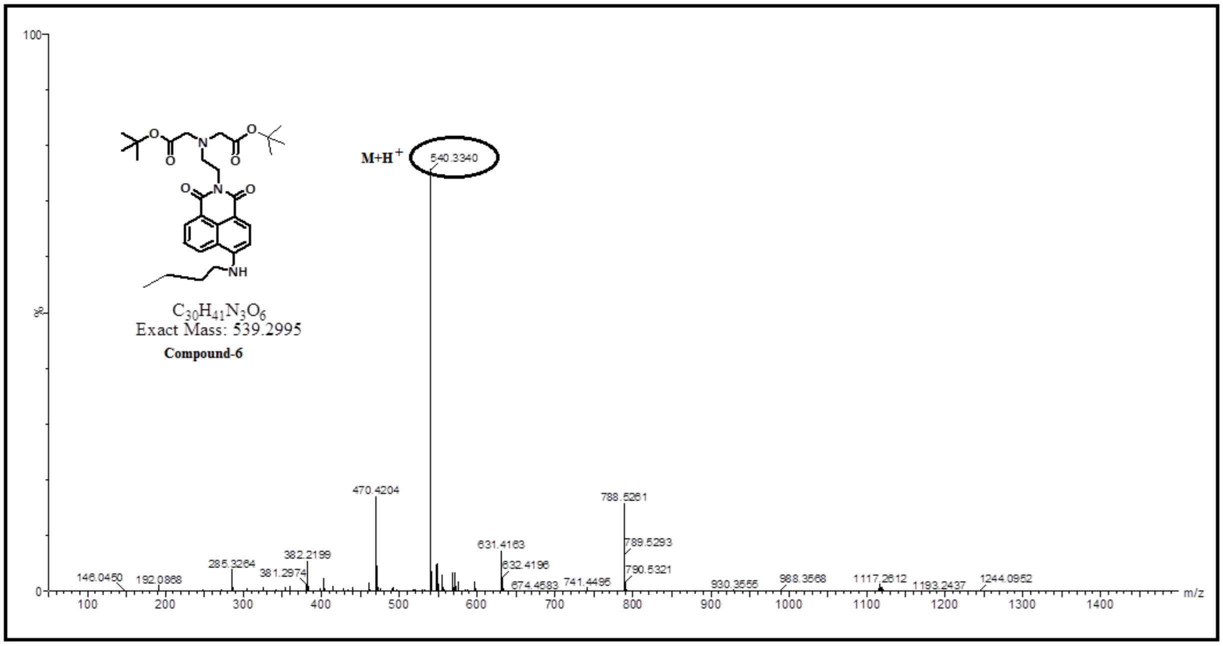

Figure S18. ESI-MS of compound **6**.

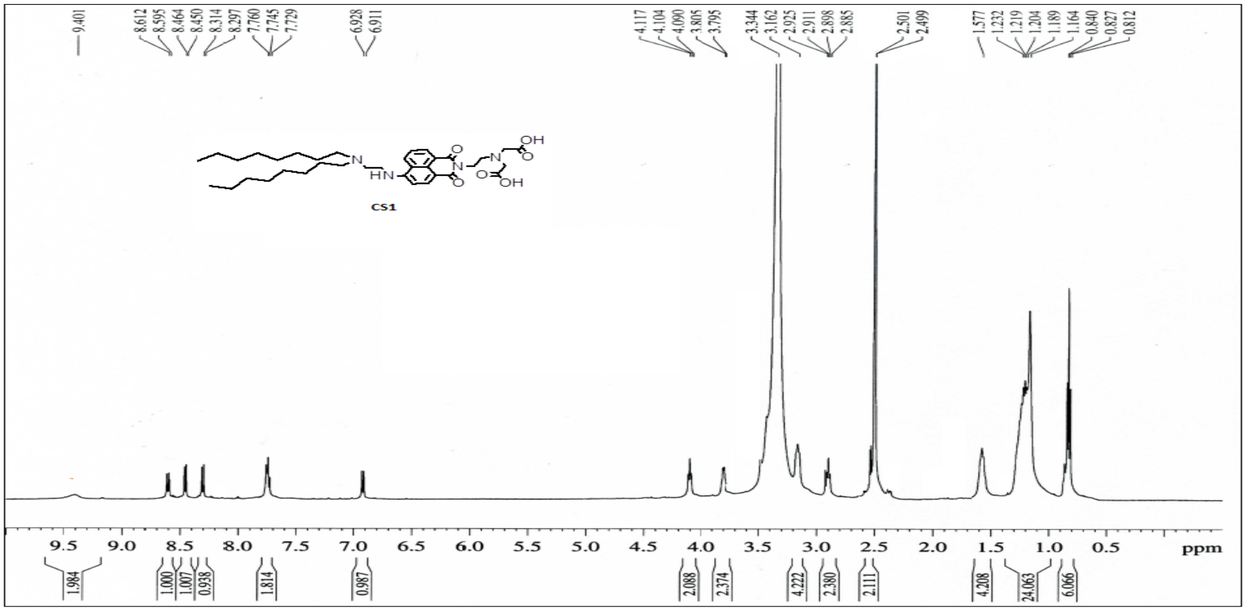

Figure S19. ^{1}H NMR spectrum of **CS-1** in DMSO-d_6.

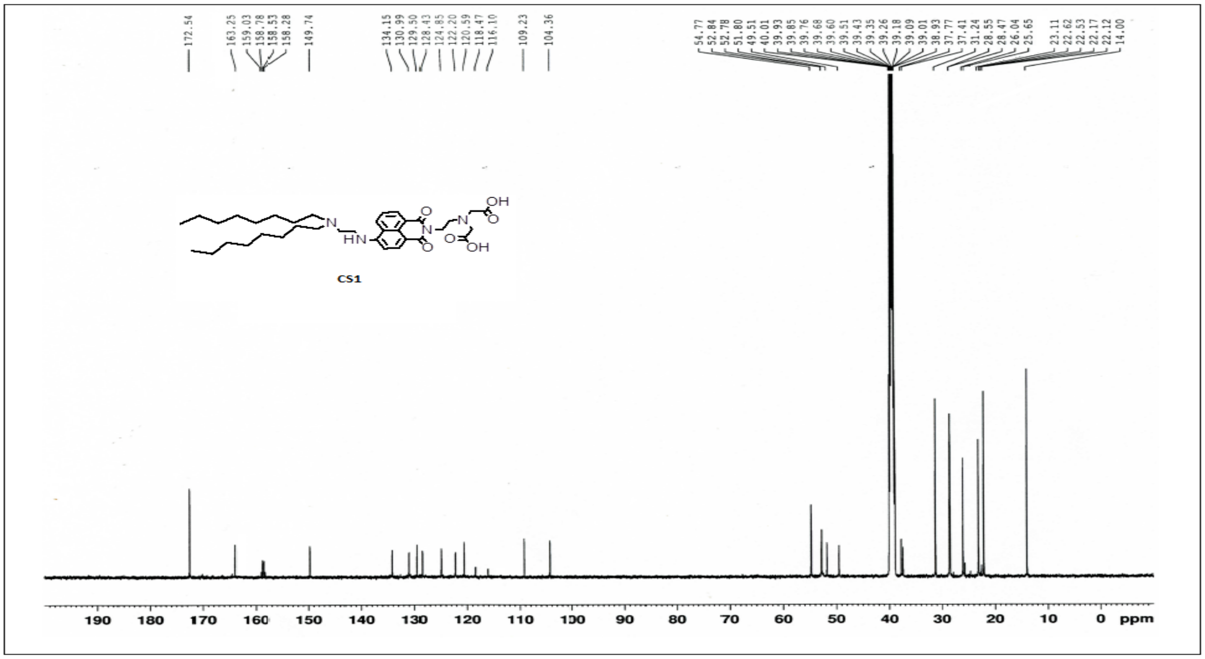

Figure S20. ^{13}C NMR spectrum of **CS-1** in DMSO-d_6.

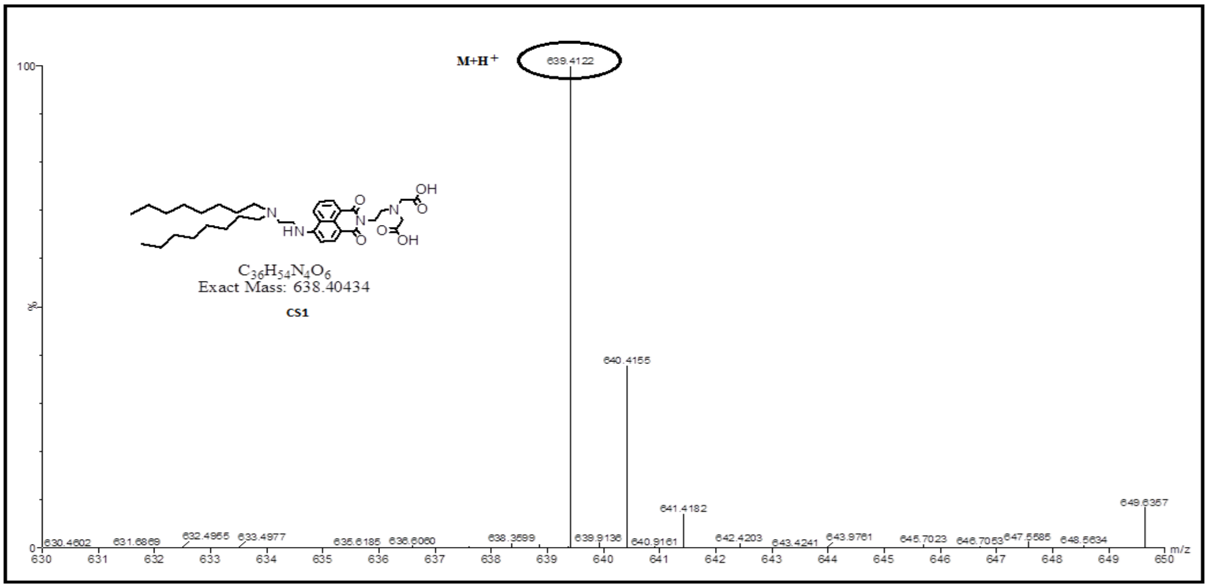

Figure S21. ESI-MS of **CS-1**.

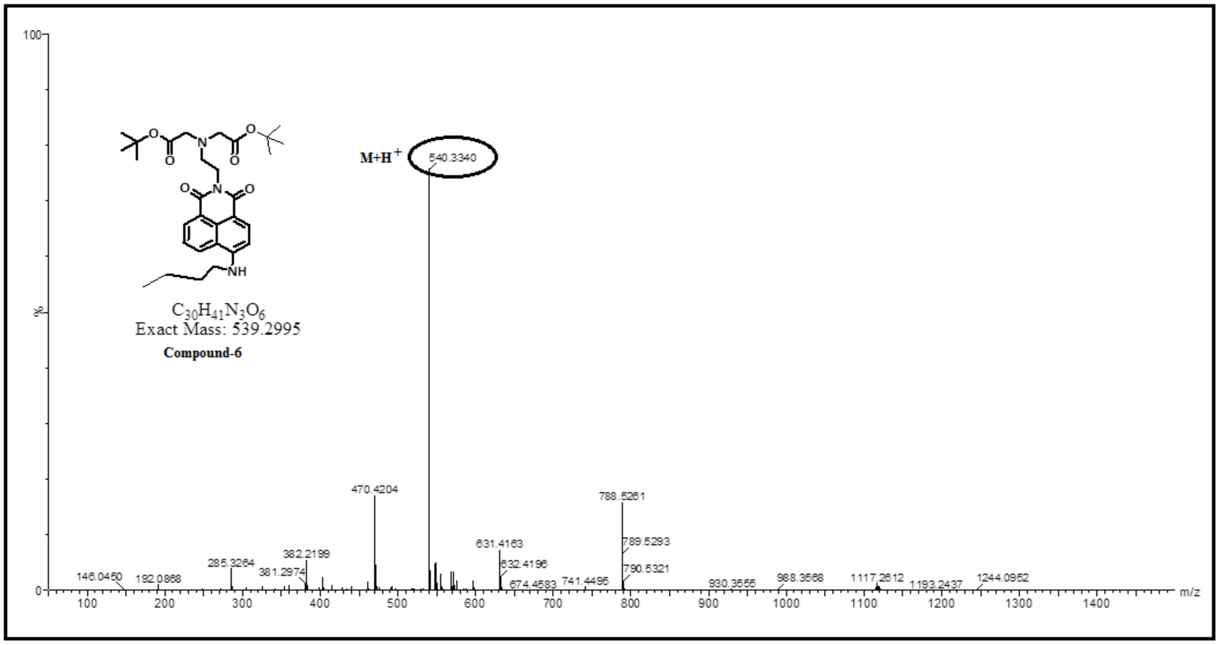

Figure S22. ^{1}H NMR spectrum of compound **R1** in DMSO-d_6.

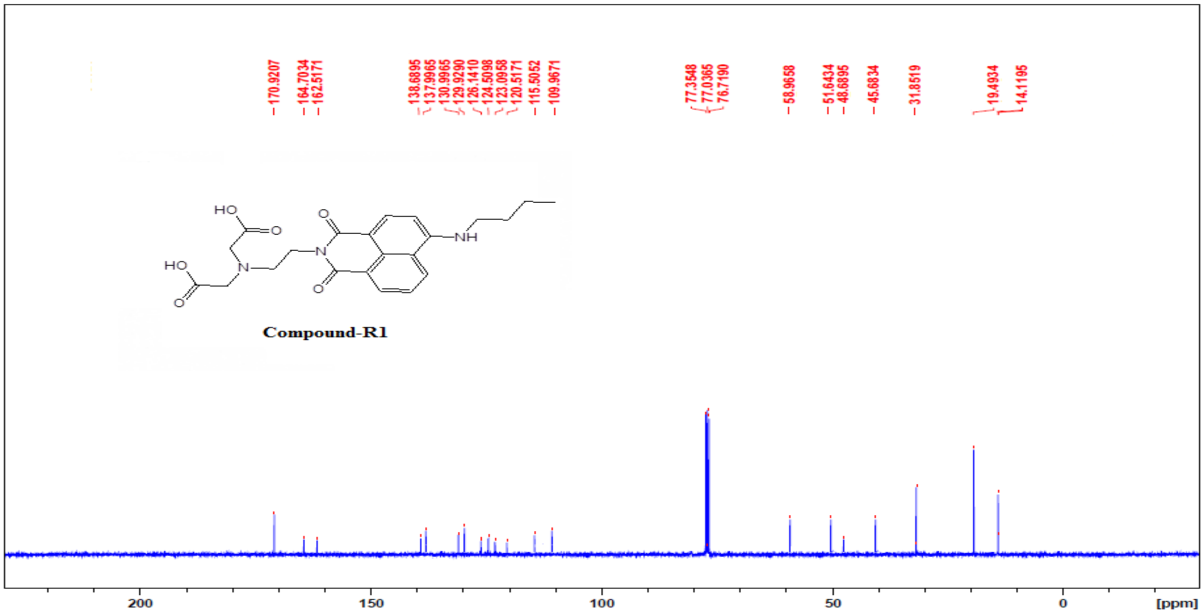

Figure S23. ^{13}C NMR spectrum of compound **R1** in $CDCl_3$.

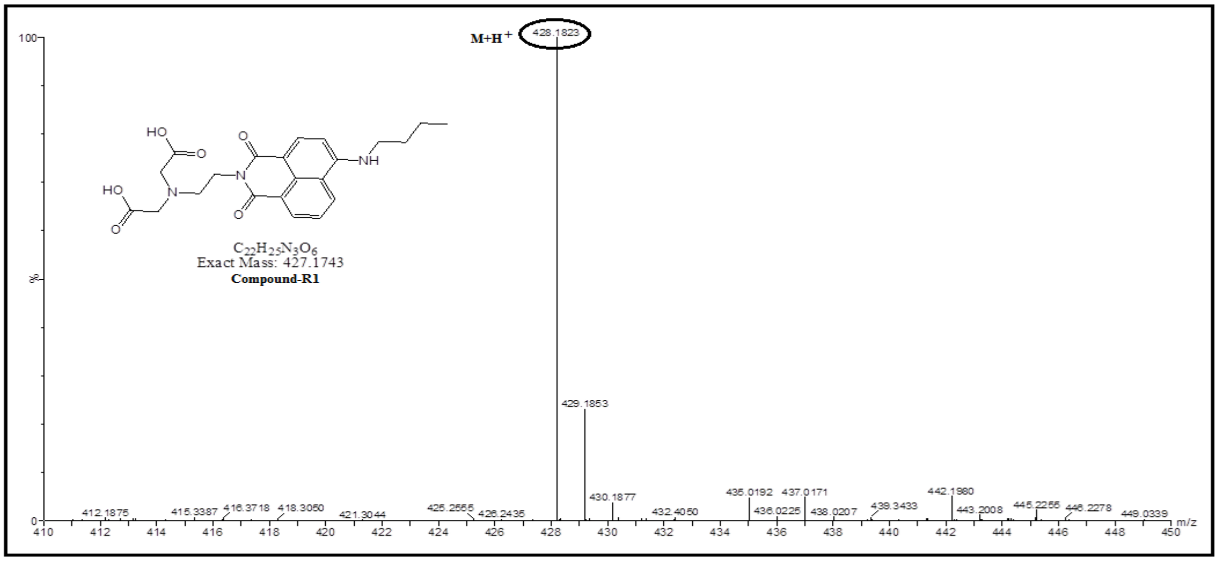

Figure S24. ESI-MS of compound **R1**.

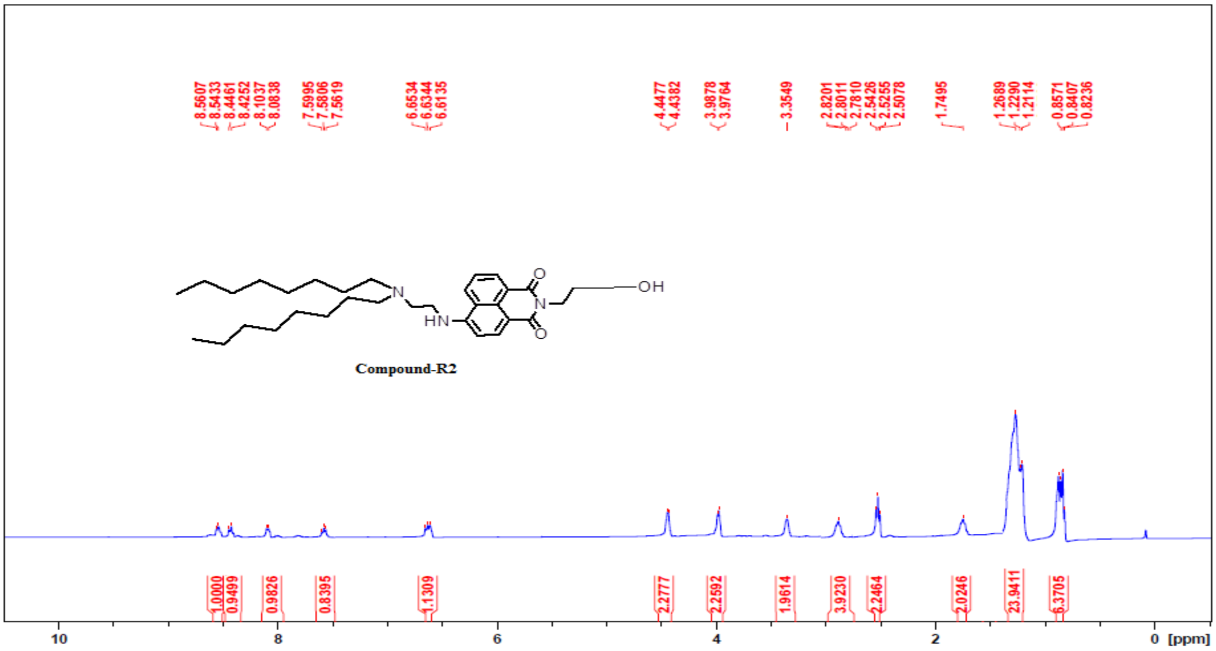

Figure S25. ^{1}H NMR spectrum of compound **R2** in $CDCl_3$.

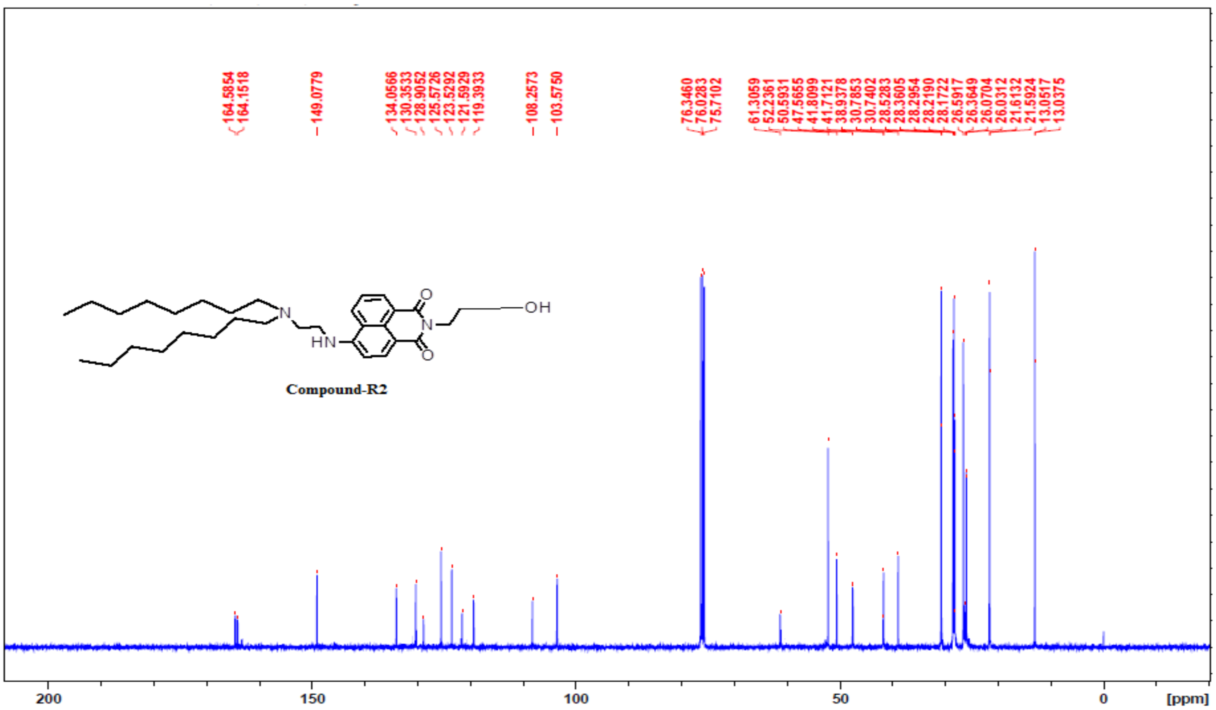

Figure S26. ^{13}C NMR spectrum of compound **R2** in $CDCl_3$.

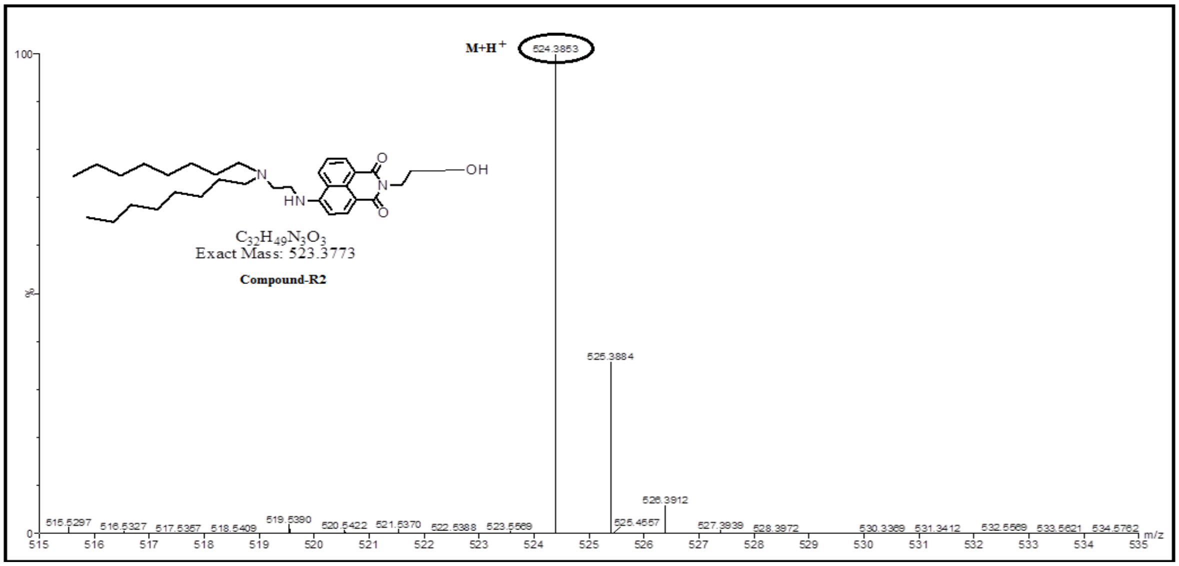

Figure S27. ESI-MS of compound **R2**.

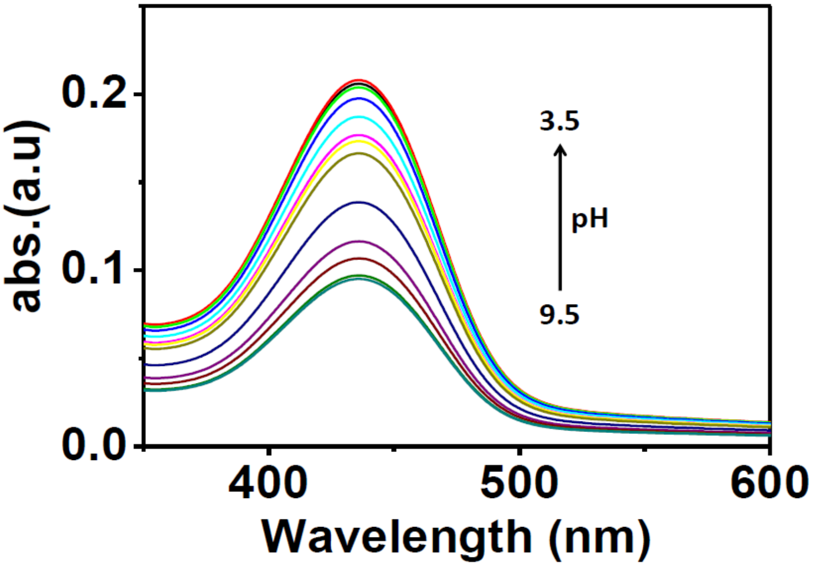

Figure S28. Changes in the UV absorption spectrum of **CS-1** (10 µM) as a function of pH (3.5–9.5) in PBS buffer containing 0.2% DMSO. **CS-1** was incubated with solutions of varying pH for 30 min at 37°C prior to recording the corresponding spectrum.

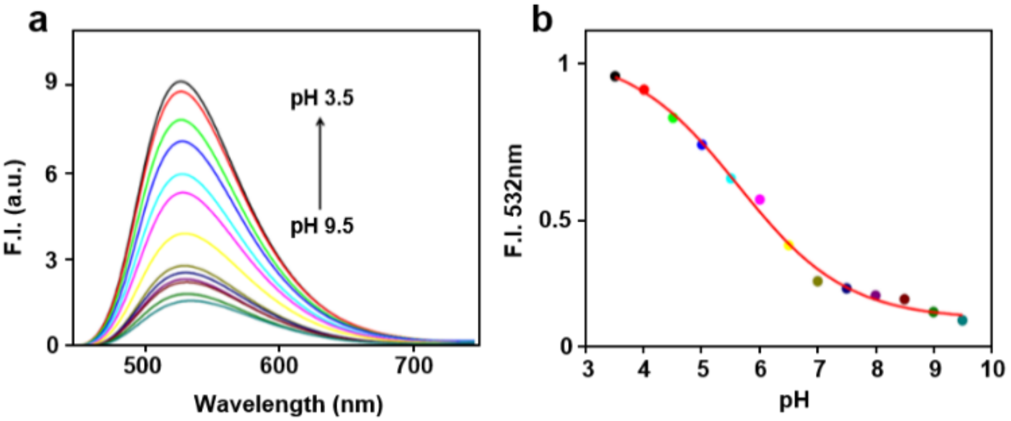

Figure S29. pH-dependent spectral features of **CS-1**. (a) Fluorescence response seen for PBS buffered aqueous solutions (0.2% DMSO) of **CS-1** (10 µM) at varying pH (3.5-9.5). (b) Relative fluorescence emission intensity at 532 nm for PBS buffered aqueous solutions (0.2% DMSO) of **CS-1** as a function of pH. At each pH, the solutions of **CS-**

1 subject to analysis were maintained for 30 min at 37°C prior to recording the individual spectra. The excitation and emission wavelengths were 440 nm and 532 nm, respectively; the slit width was 5 nm.

CS1	pH 8.5	pH 7.4	pH 6.0	pH 4.0
Quantum yield(Φs)	0.243	0.283	0.798	0.901

Figure S30. Quantum yields calculated using $\Phi s = \Phi_X (A_S F_S / A_X F_X)$ and applying this equation at pH 4, pH 6.0, pH 7.4, and pH 8.5. In all cases, excitation was effected at 440 nm.

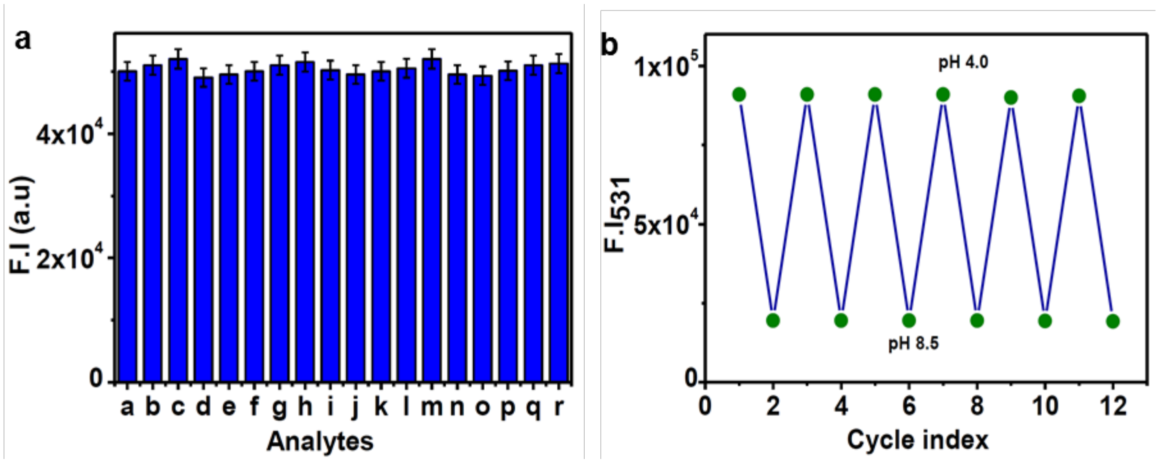

Figure S31. (a) Fluorescent emission intensities (FI_{em} = 531 nm) of **CS-1** (10 µM) recorded in the presence of 500 µM of various analytes (PBS buffer; pH 6.0): a) GSH , b) Li^+, c) Na^+, d) K^+, e) Mg^{2+}, f) Ca^{2+}, g) Fe^{2+}, h) Fe^{3+}, i) Cu^{2+}, j) Zn^{2+}, k) Cl^-, l) Br^-, m) HCO_3^-, n) NO_2^-, o) NO_3^-, p) AcO^-, q) SO_4^{2-}, r) H_2O_2. (b) Fluorescent intensity of **CS-1** (10 µM) recorded while cycling between pH 4.0 and 8.5 in PBS-buffer containing 0.2% DMSO.

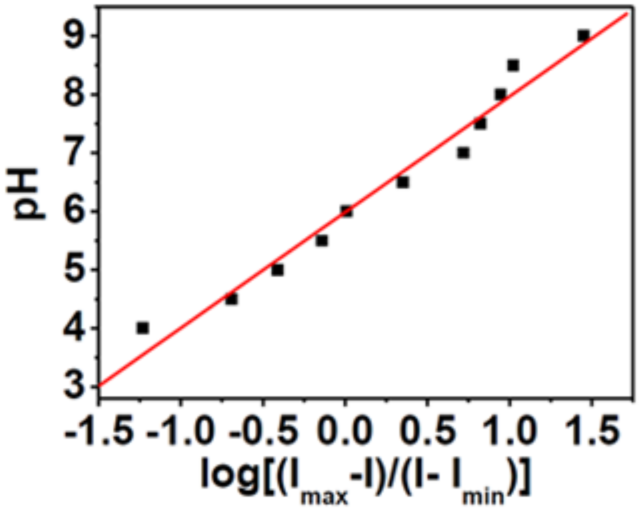

Y = A + B * X

Parameter	Value	Error
A	5.98705	0.094
B	1.98134	0.11353
R	SD<br>P	N
0.98554	0.29614<br><0.0001	11

Figure S32. Plot of pH *vs* log[(I_{max}-I)(I-I_{min})], where I is the fluorescence intensity of **CS-1** at 531 nm observed upon excitation at 440 nm. The y-intercept corresponds to the calculated pK_a value (5.987 ± 0.094) for the equilibrium between the **CS-1** and its first protonated form.

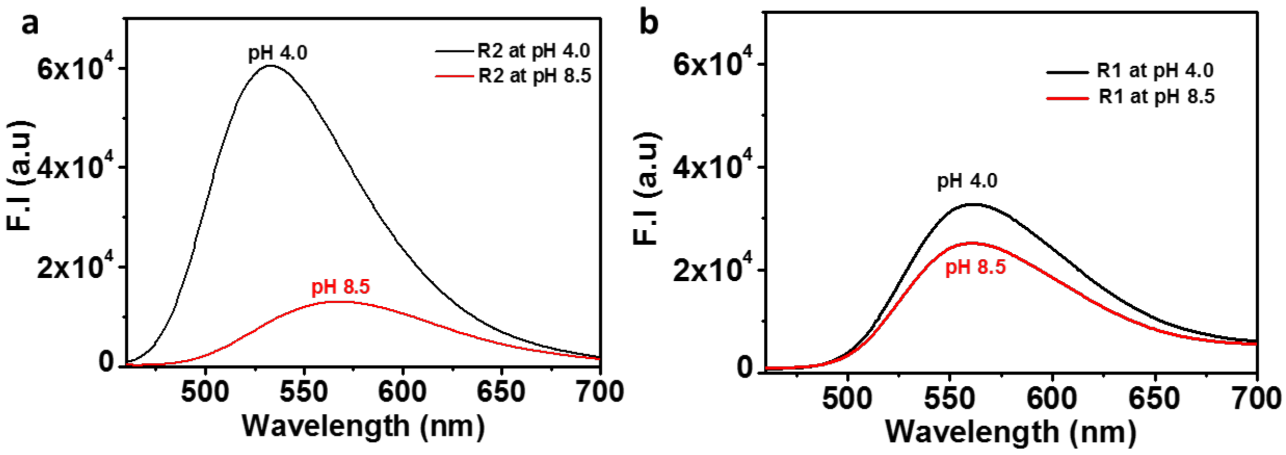

Figure S33. Changes in the fluorescence spectra of (a) **R2** and (b) **R1** (both 10 µM) at pH 4.0 and 8.5 in PBS buffer containing 0.2% DMSO. Constructs **R1** and **R2** were incubated with solutions of the indicated pH for 30 min at 37°C prior to recording the spectra. Excitation was effected at 440 nm with the excitation and emission slit widths both being set to 5 nm.

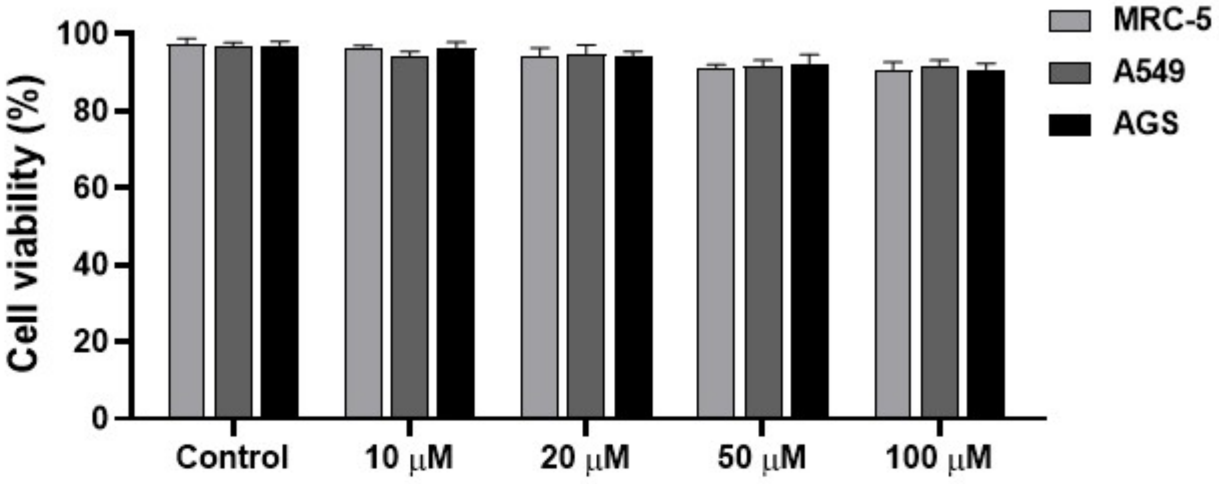

Figure S34. In vitro cell cytotoxicity of CS-1. MRC-5, A549 and AGS were treated with **CS-1** (10, 20, 50 and 100 μM) for 24 h. The cytotoxic effect of the CS-1 was measured using the CCK-8 kit (Sigma-Aldrich); study replicated in 3 independent experiments.

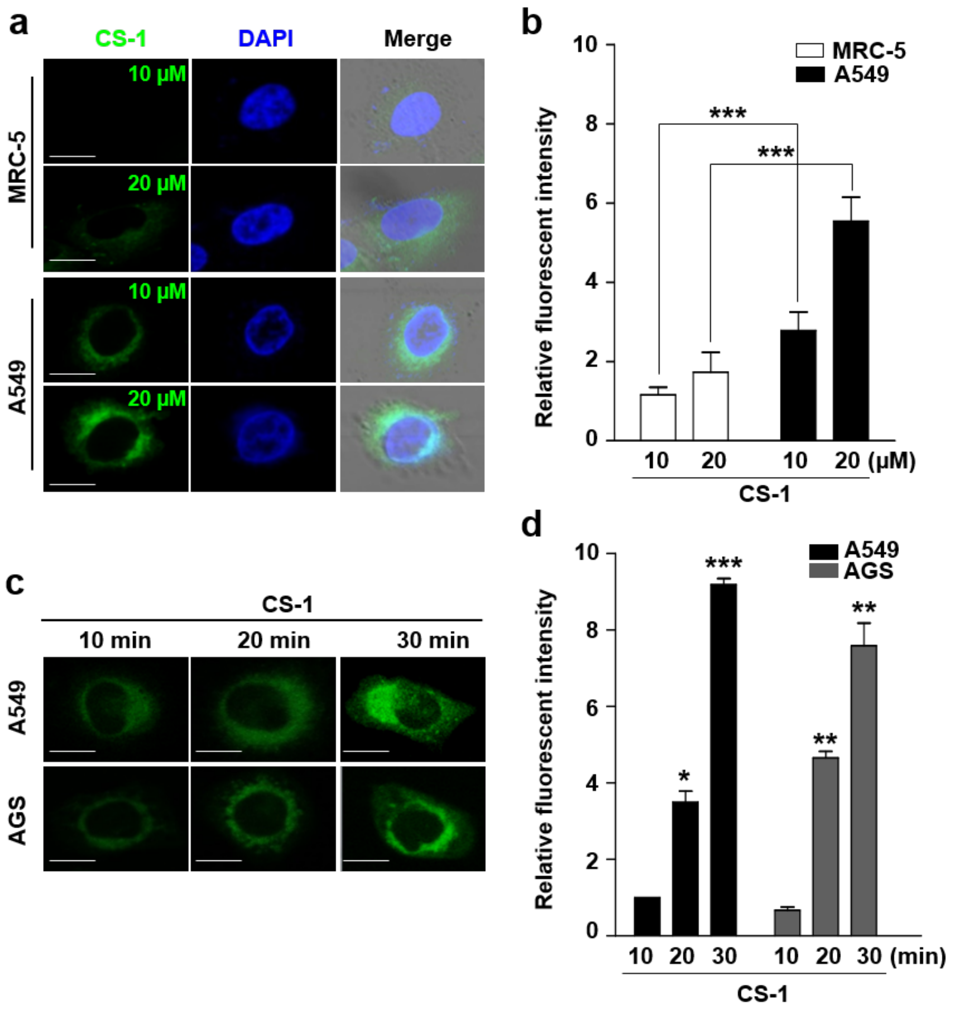

Figure S35. Fluorescence imaging of cancer and normal cells incubated in phosphate-buffered saline (at ~pH 7.4) and treated with **CS-1**. (a, b) MRC-5 normal and A549 cancer cells were treated with **CS-1** (10 and 20 μM) for 30 min. (a) Confocal fluorescence microscopic images of MRC-5 and A549 incubated with **CS-1** (green). Scale bar: 20 μm. (b) Quantification of the **CS-1** fluorescence per cell. (c, d) A549 and AGS cancer cells were treated with **CS-1** (20 μM) for the indicated times. (c) Representative fluorescence images. Scale bar: 20 μm. (d) Quantification of **CS-1** fluorescence per cell. Experiments were repeated at least 3 times. Graphs show mean ± SD. *P < 0.05, **P < 0.01, and ***P < 0.001 (two-tailed unpaired Mann–Whitney).

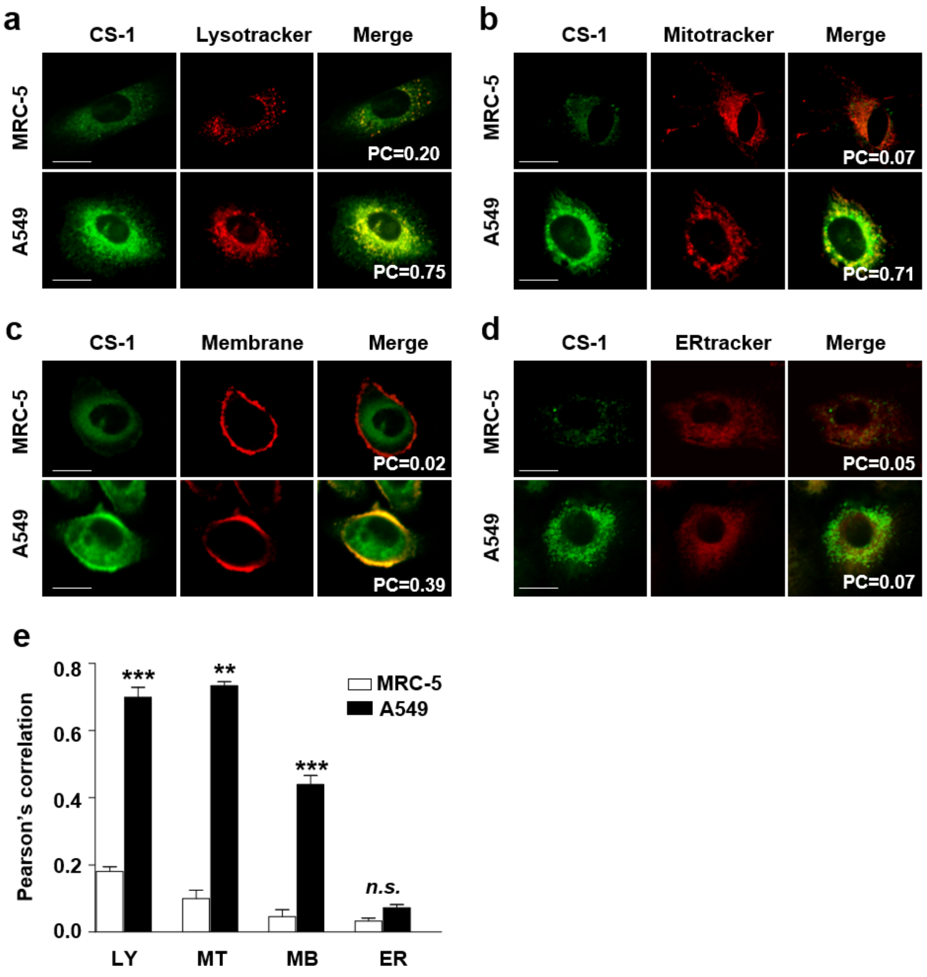

Figure S36. Fluorescence ascribed to **CS-1** in various organelles of MRC-5 normal and A549 cancer cells incubated in phosphate-buffered saline (at ~pH 7.4). (a) MRC-5 and A549 cells were co-stained with **CS-1** (20 µM) and LysoTracker® (100 nM) for 30 min. (b) MRC-5 and A549 cells were co-stained with **CS-1** (20 µM) and MitoTracker® (250 nM) for 30 min. (c) MRC-5 and A549 cells were co-stained with **CS-1** (20 µM) and CellMask™ Plasma Membrane Stain (250 nM) for 30 min. (d) MRC-5 and A549 cells were co-stained with **CS-1** (20 µM) and ER-tracker® (250 nM) for 30 min. The fluorescence of **CS-1** (green) and the lysosome, mitochondria, membrane, or endoplasmic reticulum markers (red) are shown. (e) Pearson's correlation coefficient (PC) values are shown for selected confocal images. Scale bar: 20 µm. Graphs show mean ± SD. *P < 0.05, **P < 0.01, and ***P < 0.001 (two-tailed unpaired Mann–Whitney). LY, lysosome; MT, mitochondria; MB, membrane; ER, endoplasmic reticulum.

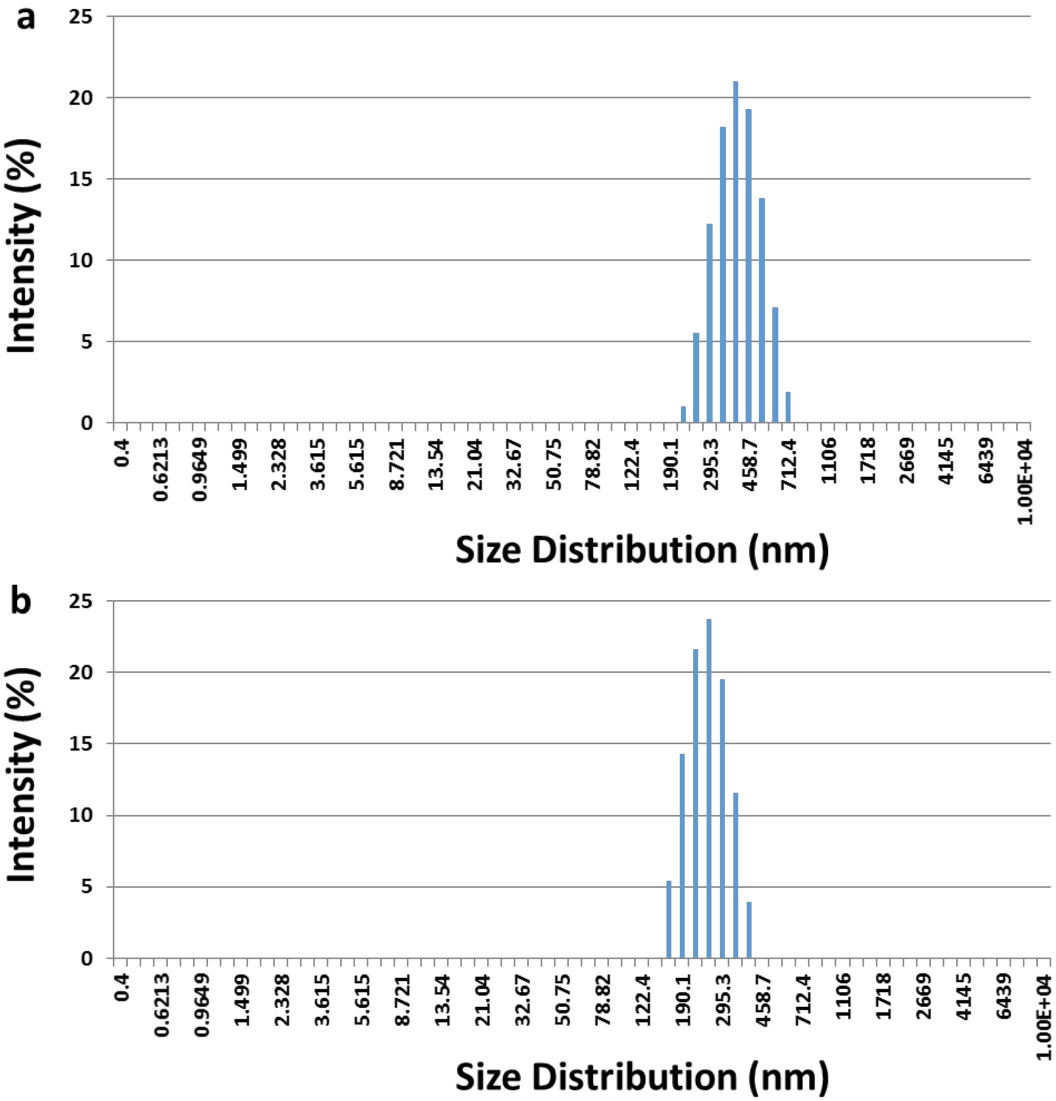

Figure S37. Results of dynamic light scattering (DLS) analyses of **CS-1** (1 mM) at pH 4.5 (a) and pH 7.4 (b). DLS measurements were performed using a Zetasizer Nano ZSP (Malvern Instruments). Measured hydrodynamic radii at pH 4.5 and pH 7.4 are 420 ± 110 nm and 260 ± 170 nm, respectively.

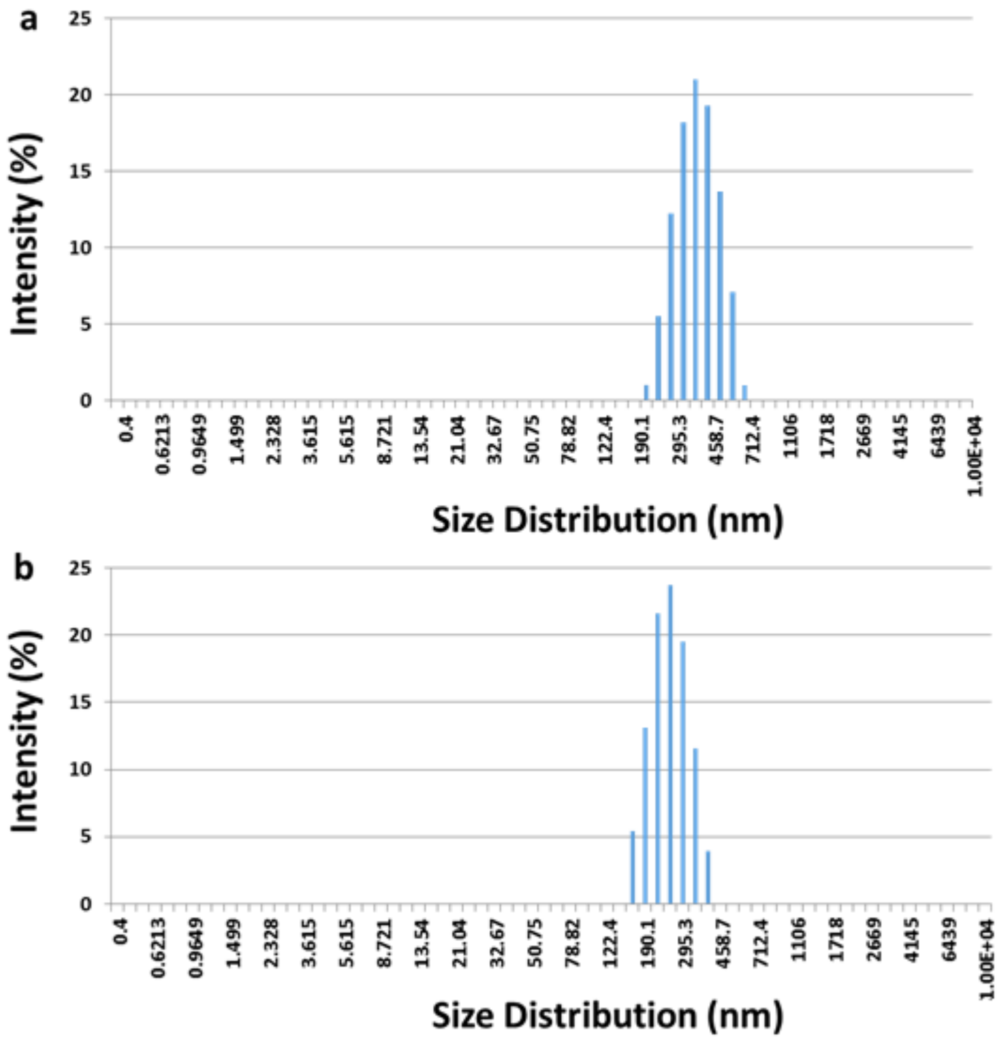

Figure S38. Stability test of **CS-1**. Results of dynamic light scattering (DLS) analyses of **CS-1** (1 mM) at pH 4.5 (a) and pH 7.4 (b) after UV irradiation for 30 minutes proved almost identical to those in Figure S36.

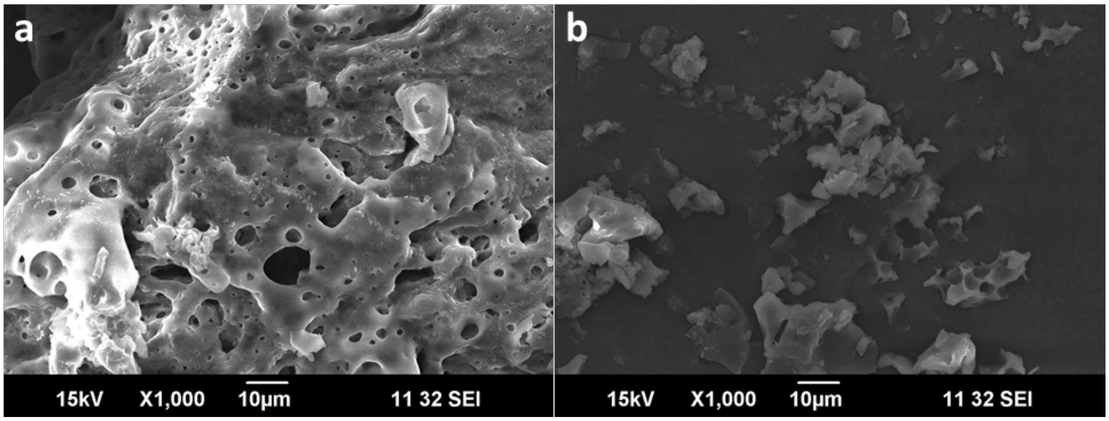

Figure S39. SEM images of **CS-1** (1 mM) at (a) pH 4.5 and (b) pH 7.4. Aqueous solutions of **CS-1** (1 mM) at the indicated pH were freeze-dried and then subject to SEM imaging on a Cu-grid using a JEOL-ASM-690-LA scanning electron microscope after Au sputtering.

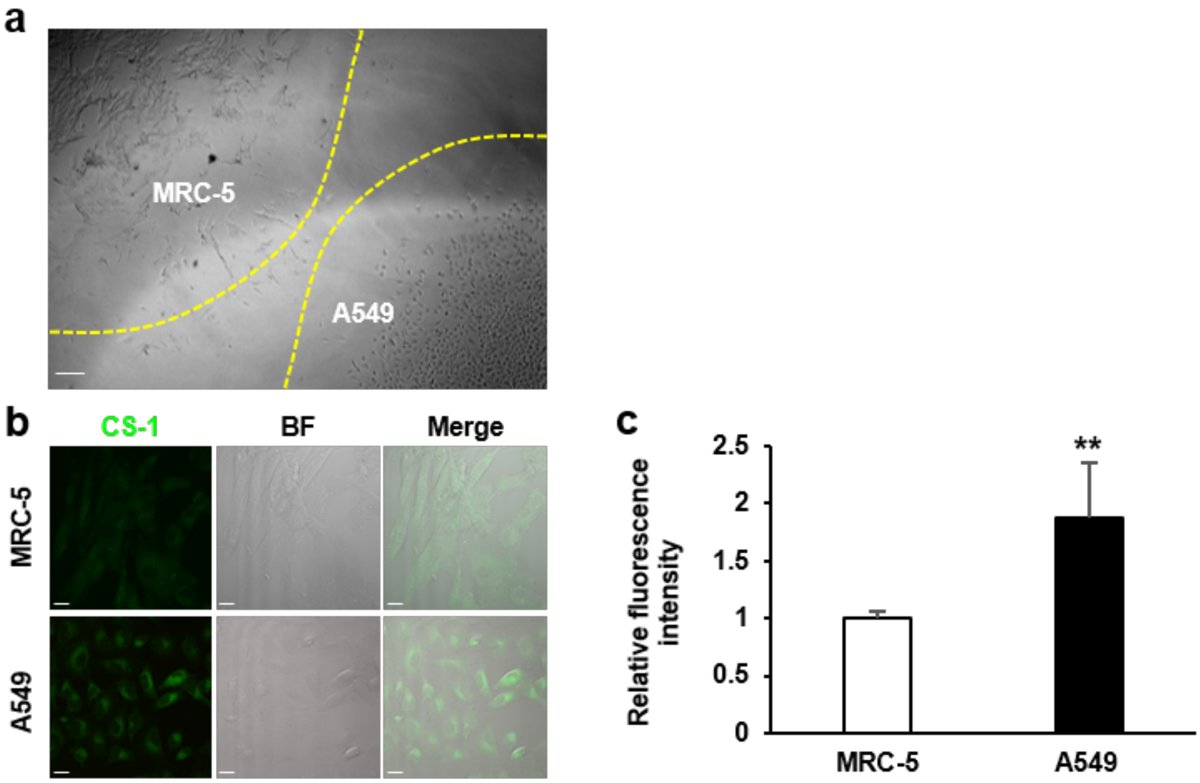

Figure S40. Fluorescence imaging of a co-culture of MRC-5 normal and A549 cancer cells incubated in phosphate-buffered saline (at ~pH 7.4) and treated with **CS-1**. The co-cultured cells were treated with **CS-1** (10 µM) for 10 min. (a) Representative bright-field (BF) image of co-cultured MRC-5 normal and A549 cancer cells. Scale bar: 200 µm. (b) Representative confocal fluorescence images of co-cultured MRC-5 and A549 cells incubated with **CS-1**. (c) Corresponding quantitative comparison of the fluorescence intensity in the co-cultured MRC-5 and A549 cells, from 6 ROIs in the cytosol area of the cells (3 independent fields). Scale bar: 20 µm. Graphs show mean ± SD. **P < 0.01 (two-tailed unpaired Mann–Whitney).

Supplementary references

(1) X. Liu, W. Zhang, C. Li, W. Zhou, Z. Li, M. Yu and L. Wei, *RSC Adv.*, **2015**, 5, 4941–4946.
